# The effects of different designs of indoor biophilic greening on psychological and physiological responses and cognitive performance of office workers

**DOI:** 10.1371/journal.pone.0307934

**Published:** 2024-07-26

**Authors:** Hiroyuki Fukumoto, Masahiro Shimoda, Saeko Hoshino

**Affiliations:** 1 Division of Environment Conservation, Institute of Agriculture, Tokyo University of Agriculture and Technology, Saiwai-cho Fuchu, Tokyo, Japan; 2 Urban Scape Unit, Green Relation Department, Greeval Co. Ltd., Minato-ku, Tokyo, Japan; Shenyang Jianzhu University, CHINA

## Abstract

Impression on biophilic designs influences the effects of indoor greening. The current study aimed to investigate the effects of different biophilic designs in office rooms on the psychological and physiological responses and the cognitive performance of office workers. Indoor greening rooms with Japanese and tropical designs were used along with the green-free (control) design in this study. The heart rate variability of the participants was not affected by green designs. However, there was improvement in impressions on tropical and Japanese designs in office rooms. In particular, the Japanese design was more effective in decreasing negative emotions than the tropical design. The electroencephalography during 5-min exposure to the greening designs showed limited frequency bands and regions of interest affected by the greenery design. Taken together with the psychological data, indoor greening with the tropical design promoted positive mood states. Meanwhile, indoor greening in the Japanese design, inhibited negative mood states. However, there were no significant differences between the two designs in terms of cognitive task performance. Hence, indoor greening increases neural efficiency during cognitive tasks.

## Introduction

In this era of global urbanization, people’s access to the natural environment is decreasing [1], and people spend most of their day indoors [2]. Decreased access to nature is associated with increased physical and mental health issues and stress-related diseases [3, 4]. Meanwhile, access and exposure to the natural environment can contribute to good physical and mental health [5]. Wilson [6] proposed the biophilia hypothesis, which states that humans are more likely to feel affection for and subconsciously seek connections with natural organisms and systems. Designing greenery based on this hypothesis is referred to as biophilic design and is attracting attention as a greening method for indoor environments (e.g., hotel lobby [7]). Indoor biophilic greening has physiological and psychological effects. That is, it lowers heart rate, blood pressure, fatigue, and mental stress. Yin et al. [8] have revealed that biophilic design elements affect stress recovery and anxiety.

Emotional and cognitive function improvements due to contact with nature were also observed [5]. Several studies have found that exposure to nature affects attention and memory. This notion is based on two theoretical perspectives of environmental psychology, which are attention restoration theory (ART) [9, 10] and stress restoration theory (SRT) [11, 12]. ART proposes that exposure to nature can replenish attentional resources and that the brain becomes fatigued due to the depletion of the directed attention mechanism in the urban environment. SRT indicates that exposure to the natural environment increases positive affect and facilitates recovery from physiological stress. Thus, lower stress levels lead to better cognitive/attentional task performance. Yin et al. [13] demonstrated reduced physiological stress and improved short-term memory in an indoor biophilic environment. The patients reported that the biophilic environment enhanced the individuals’ overall emotional statuses by decrease in negative and increase in positive emotions [13]. Although these self-reported data are promising [8, 14], more objective physiological data is required for precise quantification. As electroencephalography (EEG) signals reflect emotions, they are useful for evaluation of the effect of biophilic designs. EEG is also suitable for assessment of attention and cognition in human subjects. While the psychological effects and some physiological responses to the biophilic environment are known, its mechanisms are incompletely understood [15], partially due to researchers’ heavy reliance on behavioral measures such as questionnaires, self-assessments, and tasks in the survey methodology [16].

Changes in neural activity, particularly brain activity, must be examined before and after natural experiences to identify possible causal mechanisms for the observed effects in biophilic studies [17]. Jeong and Park [18] measured EEG and subjective affective changes elicited by visual stimulation of green plants and reported induction of physiological relaxation and positive psychological effects. Chen, He, and Yu [19] reported an association between EEG data and subjective fatigue recovery during a restorative environmental experience in a forest garden. While several biophilic studies have used EEG, some have not been able to capture the activity of the whole brain due to the small number of EEG derivation sites. Therefore, more data should be collected [18, 20].

Impressions on and preferences for the natural environment can influence cognitive processing benefits [17]. Yin et al. [21] have revealed that the effects of biophilic elements differed based on workspace type. Aristizabal et al. [22] have shown that immersive biophilic environments in office rooms can improve an occupant’s satisfaction and cognitive performance and reduce stress. A good biophilic design is based on multiple perspectives, which include the surrounding natural environment, sociocultural norms and traditions, and the function of the building [23]. It creates inspiring, restorative, healthy, and integrated spaces with the functionality of the place and the (urban) ecosystem to which it applies [24]. This study focused on the designs used in biophilic greening for office spaces. Incorporating biophilic greening into the office can be expected to reduce stress and improve cognitive performance in office workers, but previous studies commonly evaluated college students. Thus, their results cannot be directly applied to office workers. Evaluating the effects of biophilic design among people working in offices can provide direct evidence of improving office environments and achieving a better health status.

Therefore, this study aimed to assess the effects of different designs of indoor biophilic greening on stress and emotional changes and the performance of cognitive tasks using EEG among office workers. Based on ART, which focuses on the cognitive explanations of natural benefits, and SRT, which asserts the importance of emotional processes in influencing cognition, we tested the hypothesis that breaks and cognitive work in the biophilic design can improve performance and emotions. This study is unique in that all participants were healthy office workers. The effects of different designs in combination with cognitive performance and brain activity were also evaluated.

## Materials and methods

### Participants

Twenty-seven individuals (mean age: 33.4 ± 11.3 years, 14 women, 13 men) participated in this study. The participants were office workers without neurological disorders and were recruited between January 19 and February 15, 2023. Before data collection, all recruited individuals were informed about the content and aim of this study, and they provided written informed consent. This study was approved by the Ethics Committee of the Tokyo University of Agriculture and Technology (approval number no.: 221201–0440) and performed according to the recommendations of the Declaration of Helsinki.

### Greening designs for office rooms

**Fig 1:**
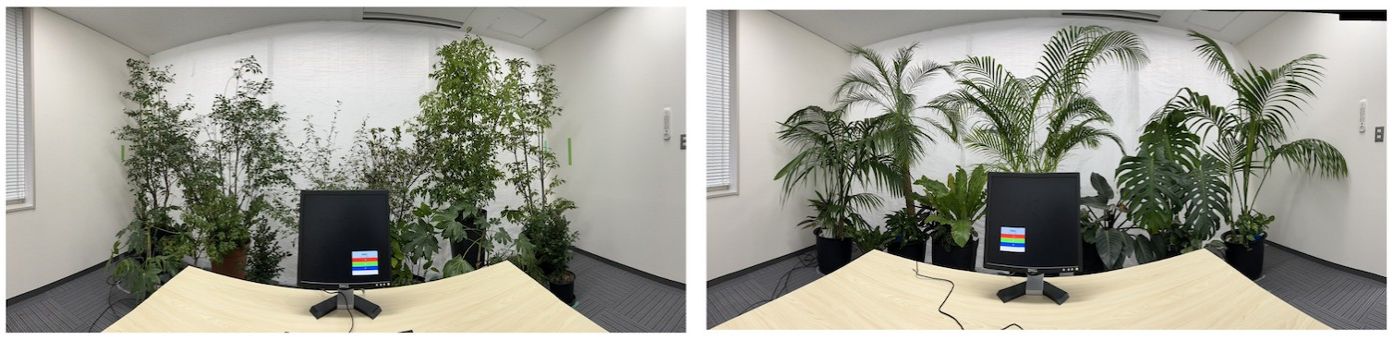
Office greening designs used in this study. Japanese (left) and tropical (right) designs.

An office room (length: 3.0 m, width: 4.0 m, and height: 2.2 m) was prepared under two greening designs (Japanese and tropical) (Fig 1) according to the conceptual framework for biophilic design [24]. The Japanese design involved greening with plants of indigenous species in Japan and East Asia, and the tropical design involved greening with tropical species (Table 1). The values of green visibility, a ratio measuring the amount of vegetation, or greenery, within a person’s field of vision, of the two designs were 0.30 and 0.31, respectively. All ornamental foliage plants were removed from the room as a control design for comparison with the greening designs. The environmental parameters of the room under the greening designs were as follows: temperature, 21.0°C ± 0.4°C; humidity, 52.6% ± 4.0%; and lux in illuminance, 430.6 ± 26.4 lx. Under the control design, the parameters were as follows: temperature, 21.1°C ± 0.4°C; humidity, 54.4% ± 2.6%; and lux in illuminance, 431.1 ± 23.0 lx.

**Table 1:** Plants used in the greening designs.

	Japanese design	Tropical design
Plants species	*Ilex pedunculosa*	*Howea belmoreana*
	*Symplocos myrtacea*	*Dypsis lutescens*
	*Eurya japonica var*. *japonica*	*Monstera*
	*Pieris japonica subsp*. *Japonica*	*Epipremnum aureum*
	*Fatsia japonica*	*Philodendron erubescens*
	*Aucuba japonica*	*Calathea orbifolia*
	*Camellia*	*Asplenium nidus*
	*Rhaphiolepis umbellata*	*Calathea ornata*
	*Polystichum polyblepharum*	*Aglaonema*
		*Cordyline fruticosa*

### Cognitive tasks

The duration of the cognitive function test was approximately 10 mins. The participants performed three consecutive tasks: Stroop color and word task (Stroop task) and 1- and 2-back tasks. All tasks were provided using a laptop computer.

#### Stroop color and word task

The name of a color was displayed on the computer screen at an interval of 1–2 s. The color of the word’s font occasionally matched the word itself (congruent condition). However, it did not sometimes (incongruent condition). The participants needed to respond the color of the word’s font (but not the word itself) before it disappeared. There were 72 trials.

#### 1- and 2-back tasks

Single digits appeared on the computer screen at 1–2 s intervals. The participants needed to determine if the current number matched the stimulus displayed *n* trials ago. Here, *n* is a variable that can be adjusted up or down to increase or decrease the cognitive load, respectively [25]. In this experiment, *n* was set to 1 (1-back) and 2 (2-back), with 48 trials.

### Outcome measurements

#### Heart rate variability

To assess autonomic nervous activity, beat-to-beat interval data of the heart were recorded using a heart rate monitor comprising a chest strap (Polar H10N, Polar Electro Oy, Finland) and a watch (Polar Unite, Polar Electro Oy, Finland). A software (Kubios HRV premium version3.5, Kubios Oy, Finland) was used to analyze the beat-to-beat interval data and calculated natural logarithm values of low (lnLF: 0.04–0.15 Hz) and high frequency (lnHF: 0.15–0.4 Hz) components of heart rate variability (HRV), LF/HF value, and heart rate (HR).

#### EEG (theta, alpha, beta power)

An electroencephalogram (EEG) was used with a 32-ch wireless EEG headset (Emotiv Flex, Emotiv, the USA). Thirty-two gel electrodes were attached on the positions of the international 10/20 system (FP1, FP2, F3, Fz, F4, F7, F8, FC1, FC2, FC3, FC4, FT9, FT10, C3, Cz, C4, T7, T8, CP1, CP2, CP5, CP6, TP9, TP10, P3, Pz, P4, P7, P8, O1, Oz, and O2). The sampling rate was 128 Hz. EEGLAB for MatLab© [26] was used for the offline processing of the EEG data. The power line noise was removed using the EEGLAB’s CleanLine plug-in. An independent component analysis was used to correct eye movements. Channels containing excessive artifacts were interpolated. Brain activity was assessed as five frequency bands: theta (4–8Hz), alpha 1 (8–10 Hz), alpha 2 (10–13 Hz), beta 1 (13–20 Hz), beta 2 (20–30 Hz). Power spectrum analysis of EEG signals in each frequency band can show absolute and relative powers. The absolute power of an EEG signal is the sum of the power spectral density values for each frequency band of the EEG signal. Relative power is the percentage of power in a given band compared to the total power of the EEG signal in each emotion. Meta-analyses and systematic reviews have reported that the frequency components of EEG reflect mental state, physiological function, and cognitive workload [27, 28]. Generally, the lower and upper alpha rhythms signify relaxation and alertness, respectively, whereas the beta rhythm indicates thinking, concentration, tension, and anxiety.

Previous studies have used relative theta power to indicate relaxation and relative beta 2 power to indicate stress state [20, 29]. To explore the relationship between EEG frequency and mental state and cognitive workload [28], relative values of each frequency were calculated from the absolute value using the formula: Relative value = [Absolute value] / [Total (4–50Hz) value]. EEG signal data from 32 sites were organized into five regions of interest (ROIs): ROI-1, left-frontal (FP1, F3, F7), ROI-2, right-frontal (FP2, F4, F8), ROI-3, left-posterior (P3, P7, O1), ROI-4, right-posterior (P4, P8, O2), ROI-5, midline (Fz, Cz, Pz).

#### Assessment of space impression using the semantic differential method

The participants rated their impressions of the room spaces with greening using biophilic greening designs (Japanese, tropical) and without greening (control) using the semantic differential method (SDM). The SDM is a 7-point Likert scale (1–7) consisting of 10 adjectives: uncomfortable–comfortable, restless–calm, quiet–noisy, unfriendly–friendly, dark–blight, tightness–spacious, dislike–like, Western–Japanese style, not healed–healed, and artificial–natural. High scores indicated a good impression of the space.

#### Positive and negative affect schedule

The Positive and Negative Affect Schedule (PANAS) Japanese edition was used because it is reliable, valid, easy to implement, and can be used in previous biophilic studies [30]. The participants rated the extent to which they currently felt 16 moods (8 positive and 8 negative) on a 6-point Likert scale ranging from 1 (very slightly) to 6 (extremely).

#### Cognitive performance (reaction time and accuracy of responses) in cognitive tasks

All cognitive task performances were evaluated in terms of accuracy rate (percentage of correct responses) (%) and reaction time (ms). For the Stroop task, the percent correct and reaction time were calculated for the congruent and incongruent conditions, respectively.

### Procedures

**Fig 2:**
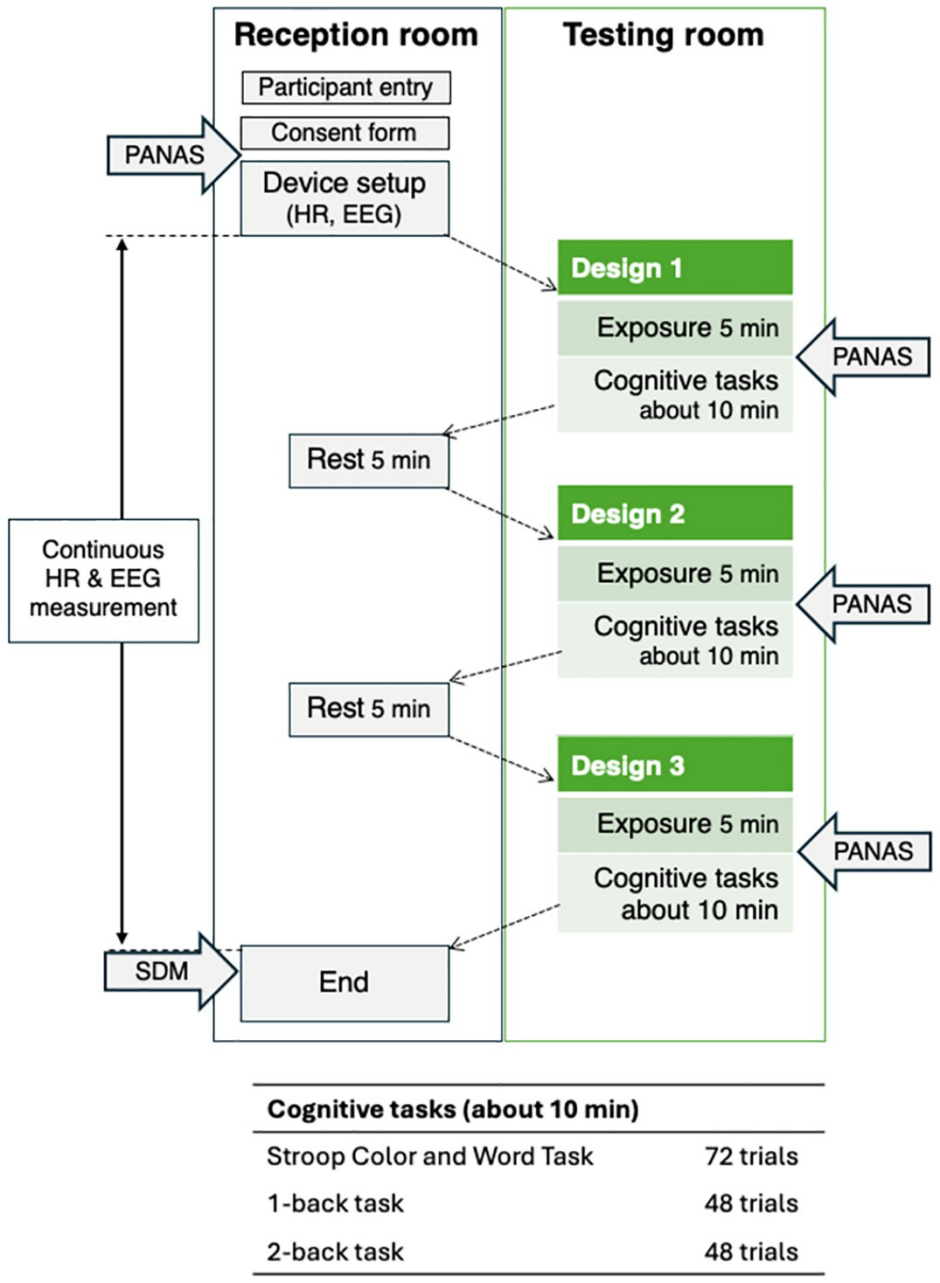
Experimental procedure. Participants consecutively performed Stroop Color and Word Task, 1-back, and 2-back tasks in the cognitive task. The order of designs 1, 2, and 3 was balanced between participants. HR, heart rate; EEG, electroencephalography; PANAS, Positive, and Negative Affect Schedule; SDM, semantic differential method.

Fig 2 shows the experimental procedure in this study. The participants sat on a chair and rated their mood using PANAS in a reception room. After attaching the HR monitor and EEG electrodes, the participants were moved to the testing room arranged with one of three designs. Next, as an experimental session, the participants sat on a chair for 5 min (5-min exposure), similar to when taking a break during work, and they were asked to rate their mood using PANAS. Next, they performed the cognitive task for approximately 10 mins and moved to the reception room to take a break for 5 min. The experimental session, which lasted for approximately 20 min, was repeated under the Japanese, tropical, and control designs. The order of the designs was balanced between participants. After the last session, the participants were asked to rate their impressions of the three designs using the SDM. Beat-to-beat interval data of the heart and EEG were recorded continuously during the experiment.

### Statistical analysis

Nine participants’ data were excluded because of recording issues (e.g., too much EMG, EOG, and baseline fluctuations on EEG) and behavioral problems (e.g., over ± 3 standard deviation errors in cognitive tasks). The remaining data from the 18 participants were used for statistical analysis.

In evaluating space impressions with the SDM, the mean value was calculated for each of the 10 pairs of adjectives for each space. One-way repeated measure analysis of variance (ANOVA) was performed using the means obtained, and each item was compared between designs (control, Japanese, and tropical). One-way repeated measure ANOVA was conducted to determine if there were differences between the baseline, control, Japanese, and tropical conditions for the positive affect (PA) and negative affect (NA) of the PANAS, respectively. To examine the extent of the change in PA and NA resulting from different greenery designs, differences relative to the control design were calculated in Japanese and tropical designs, respectively, and compared using paired t-test. The accuracy rate and reaction time in the Stroop task were analyzed using a two-way repeated measure ANOVA of design (control, Japanese, and tropical) × the task condition (congruent, incongruent). One-way repeated measure ANOVA was conducted to determine whether accuracy rate and reaction time differed between the designs (control, Japanese, and tropical) in the 1- and 2-back tasks. For the HRV data (lnHF, LF/HF, and HR) of the three designs, the value during the 5-min exposure and the averaged values of the three cognitive tasks were used. We examined whether there were differences between the designs (control, Japanese, and tropical) and between tasks (5-min exposure, approximately 10-min cognitive task). EEG data were statistically processed for each 5-min exposure, Stroop task, 1-back task, and 2-back task. One-way repeated measure ANOVA was performed to assess differences in absolute and relative powers for the five frequency bands (theta, alpha 1, alpha 2, beta 1, and beta 2) between the designs (control, Japanese, and tropical) for each ROI. The Bonferroni’s multiple comparison test was performed as a post-hoc analysis if the ANOVA results had a main effect. All statistical analyses were performed using the Statistical Package for the Social Sciences software version 24 (SPSS Inc., Chicago, IL, the USA) and Jamovi version 2.3 [31]. A *p-value* of <0.05 was considered statistically significant.

## Results

### Psychological indices

**Fig 3:**
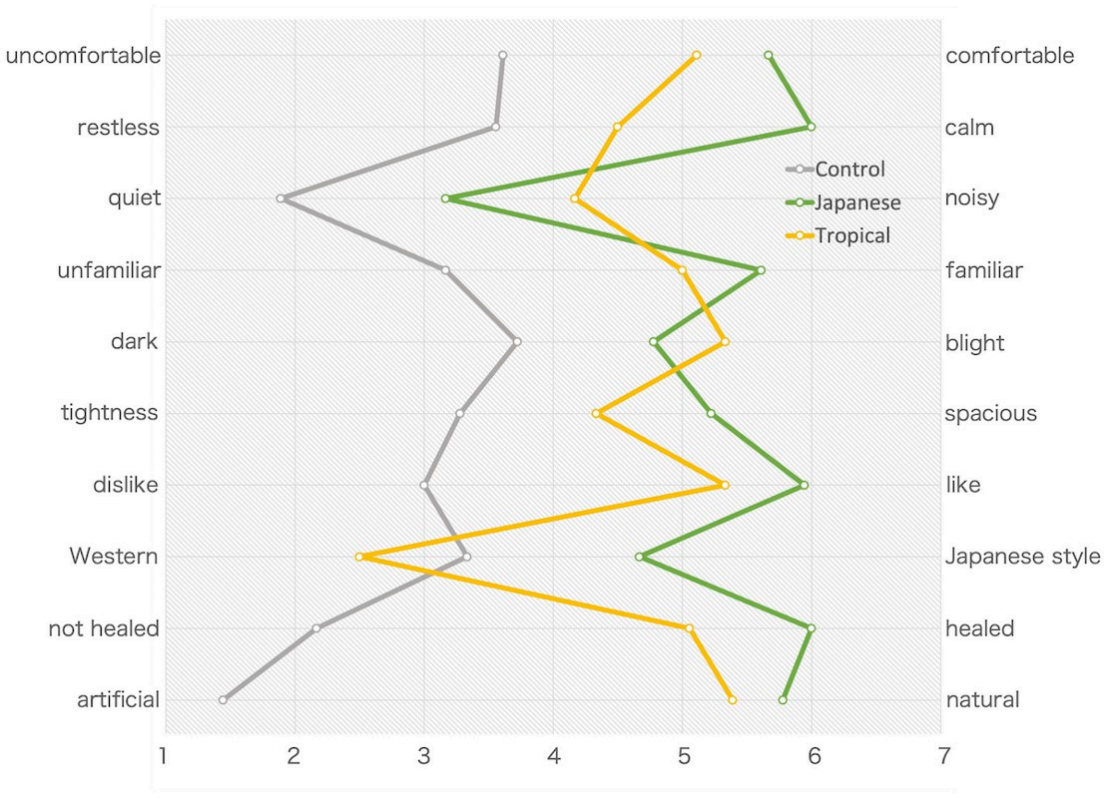
Space impression evaluated using the SDM (7-point Likert scale). Japanese and tropical designs with high scores indicated a good space impression. Control, control design; Japanese, Japanese design; Tropical, tropical design.

Greening designs (except for the Western–Japanese style item) had a higher mean value for each space impression using the SDM. The mean values of the Japanese condition were significantly higher than those of the tropical condition for items such as uncomfortable–comfortable, restless–calm, Western–Japanese, unfamiliar–familiar, tightness–spacious, and not healed–healed (Fig 3 and S1 Table).

The main effect of the condition was observed in the mean PA value in PANAS [*F* (3, 51) = 8.18, *p* < 0.01, partial η^2^ = 0.33]. Bonferroni’s multiple comparison tests between designs showed that the mean of the baseline was higher than that of the control (*p* < 0.01) and Japanese designs (*p* = 0.04). However, they were not affected by the greening conditions. A paired t-test revealed that increment in PA (ΔPA) were same in Japanese and tropical designs (p = 0.61) (Table 2). The main effect of the condition was also observed in NA [*F* (2, 34) = 6.53, *p* < 0.01, partial η^2^ = 0.28]. Bonferroni’s multiple comparison tests between designs showed that the mean of the Japanese design was lower than that of the control (*p* < 0.01) and tropical designs (*p* = 0.02). Paired t-test revealed that decrement in NA (ΔNA) were larger in Japanese than tropical designs (p < 0.01) (Table 2).

**Table 2:** Positive affect (PA) and negative affect (NA) at baseline and under the control, Japanese, and tropical designs.

	PA		NA	
	Mean	SD	Mean	SD
Baseline	22.83	4.78	15.83	4.97
Control	17.56	5.81	16.39	6.43
Japanese	18.94	6.63	13.11	4.1
Tropical	19.50	6.64	15.28	4.71
*F*-value	(3, 51) = 8.18		(3, 51) = 5.66	
Partial η^*2*^	0.33		0.25	
*P*-value	***< 0*.*01***		***< 0*.*01***	
Post-hoc	***Baseline > Control (p < 0*.*01)*;**		***Control > Japanese (p < 0*.*01)*;**	
	***Baseline > Japanese (p = 0*.*04)***		***Tropical > Japanese (p = 0*.*02)***	
	Δ PA		Δ NA	
	Mean	SD	Mean	SD
Japanese	1.39	4.82	-3.28	3.97
Tropical	1.94	3.33	-1.11	4.67
*t*-value	(17) = -0.52		(17) = -3.15	
*P*-value	0.61		***< 0*.*01***	
			***Tropical > Japanese***	

Values were expressed as mean and standard deviation.

Bold and italic indicates statistically significant

Control, control design; Japanese, Japanese design; Tropical, tropical design; SD, standard deviation; ΔPA and ΔNA; the differences against control design in PA and NA, respectively

### Physiological indices

#### HRV

The HRVs were analyzed using the two-way repeated measure ANOVA of design (control, Japanese, and tropical) × task (5-min exposure, cognitive task) (S2 Table). The designs did not have significant effects on the HRV. The tasks significantly affected LF/HF [*F* (1, 17) = 8.79, *p* = 0.009, partial η^2^ = 0.341] and HR [*F* (1, 17) = 5.46, *p* = 0.032, partial η^2^ = 0.243], but not lnHF. That is, LF/HF in cognitive task was lower than 5-min exposure, and HR in cognitive task was higher than 5-min exposure.

#### EEG

One-way ANOVA was performed on each frequency in the 5-min exposure, Stroop task, 1-back task, and 2-back task (S3–S7 Tables).

**Fig 4:**
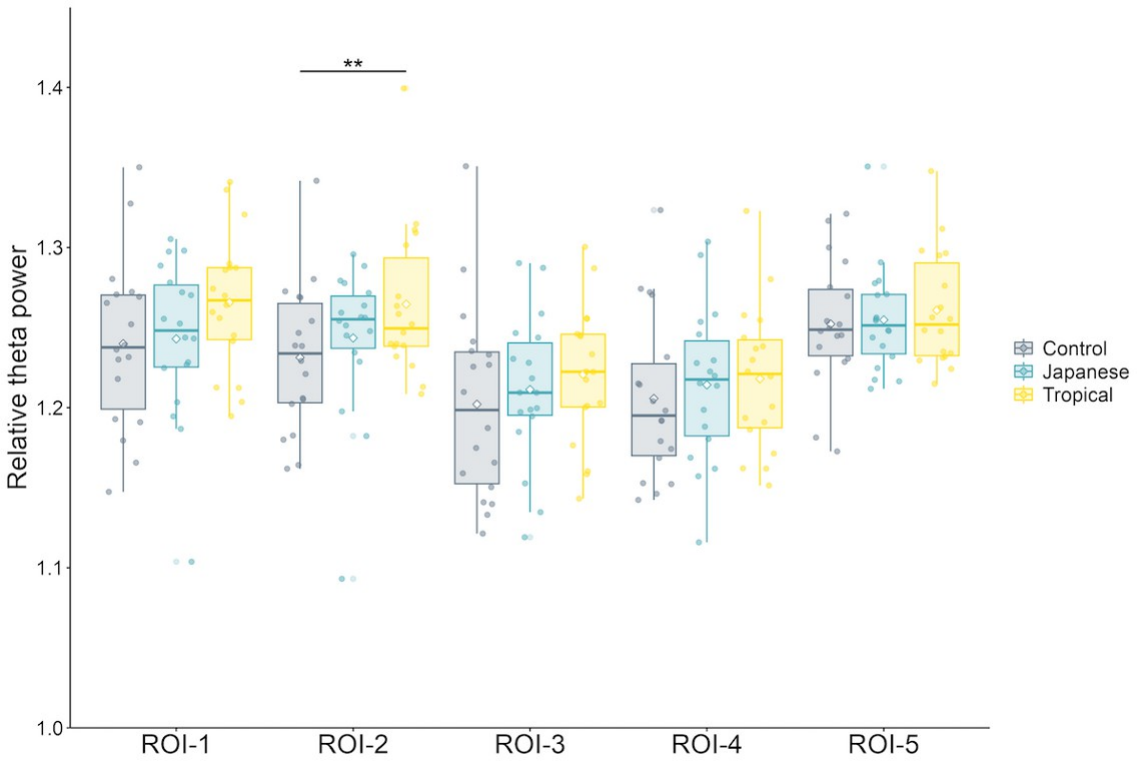
Comparison of the relative theta power according to design in the 5-min exposure. The tropical design had significantly higher relative theta power in ROI-2 than the control design (*p* < 0.01). Error bars are expressed as standard deviation. Asterisks above the bars indicate statistically significant between-design differences (**: *p* < 0.01). ROI, Region of interest; Control, control design; Japanese, Japanese design; Tropical, tropical design.

*Theta power*. In the 5-min exposure, the design had a significant effect on relative theta power in ROI-2 alone [*F* (2, 34) = 0.58, *p* < 0.01, partial η^2^ = 0.26]. As shown in Fig 4, in the post-hoc test, the tropical design had a significantly greater relative theta power than the control design (*p* < 0.01). In the Stroop task, the design significantly affected absolute theta power in ROI-2 [*F* (2, 34) = 3.87, *p* = 0.03, partial η^2^ = 0.19]. Nevertheless, the post-hoc test did not show significant differences.

*Alpha power*. In the Stroop task, the designs had a significant effect on absolute alpha 1 power in ROI-2 [*F* (2, 34) = 5.96, p < 0.01, partial η^2^ = 0.26]. In the post-hoc test, the tropical design had a significantly higher absolute alpha 1 power than the control design (*p* < 0.01) (S4 Table). In the 2-back task, the design had a significant effect on absolute alpha 1 power in ROI-5 [*F* (2, 34) = 4.48, *p* = 0.02, partial η^2^ = 0.21]. In the post-hoc test, the tropical design had a significantly higher absolute alpha 1 power than the Japanese design (*p* = 0.02) (S4 Table).

The design had significant effects on absolute alpha 2 power in ROI-1 and ROI-2 and ROI-4 in the 5-min exposure [ROI-1: *F* (2, 34) = 4.08, *p* = 0.03, partial η^2^ = 0.19; ROI-2: *F* (2, 34) = 3.56, *p* < 0.04, partial η^2^ = 0.18; and ROI-4: *F* (2, 34) = 3.41, *p* = 0.05, partial η^2^ = 0.17]. In the post-hoc test, the control design had a significantly higher absolute alpha 2 power in ROI-4 alone than the tropical design (*p* < 0.049) (S5 Table). The design significantly affected the Stroop task’s absolute alpha 2 power in ROI-2 [*F* (2, 34) = 4.38, *p* = 0.02, partial η^2^ = 0.21]. In the post-hoc test, the tropical design had a significantly higher absolute alpha 2 power than the control design (*p* = 0.04) (S5 Table).

**Fig 5:**
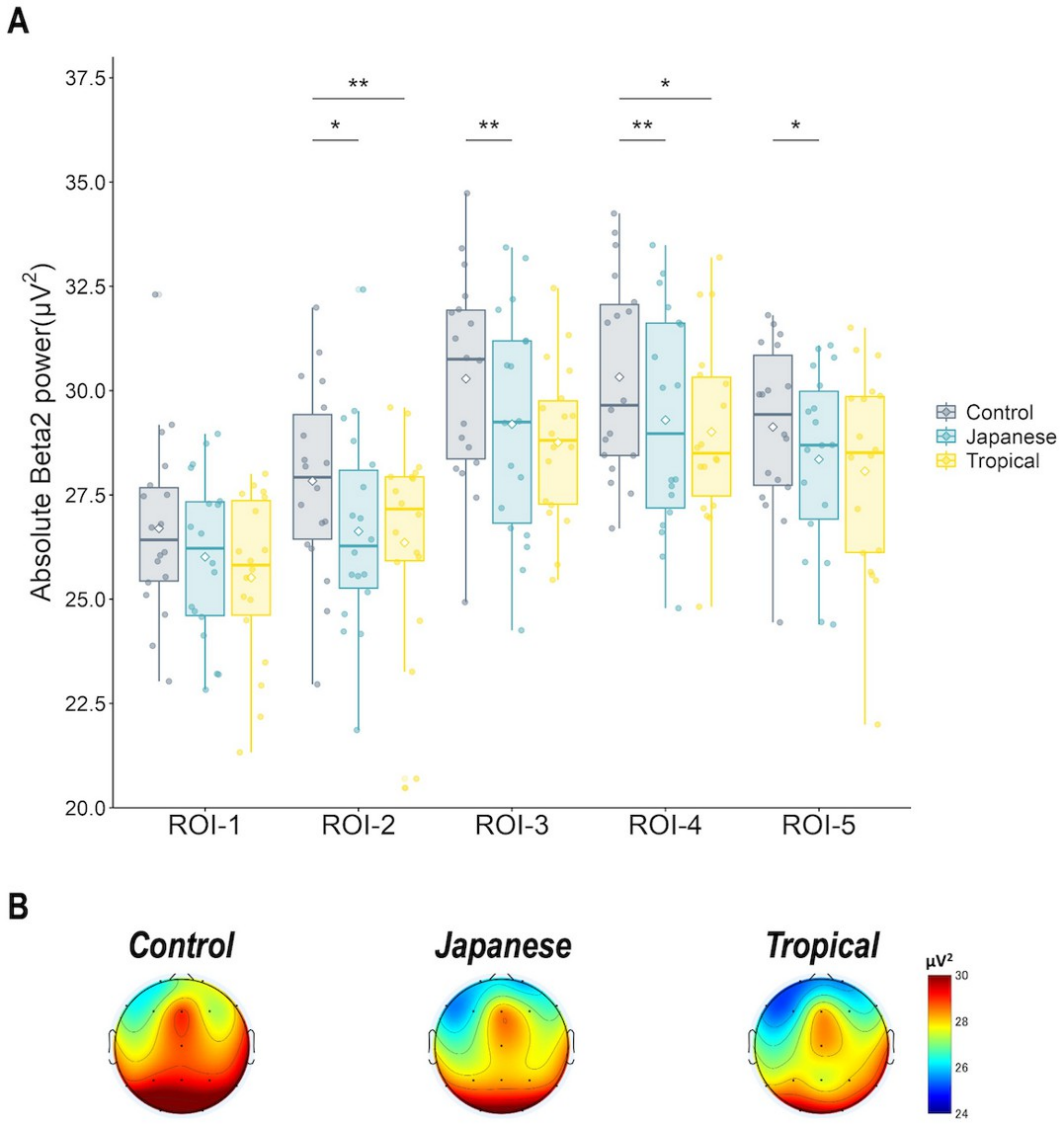
(A) Absolute beta 2 power chart and corresponding (B) topograms according to design in the 5-min exposure. Absolute beta 2 power in all ROIs except ROI-1, the control design was significantly higher than that of the greening design. Error bars are expressed as standard deviation. Asterisks above the bars indicate statistically significant between-design differences (*: *p* < 0.05; **: *p* < 0.01). ROI, Region of interest; Control, control design; Japanese, Japanese design; Tropical, tropical design.

*Beta power*. There were no statistically significant differences among the designs in the absolute beta 1 power and relative beta 1 power. In the 5-min exposure, the design had a significant effect on absolute beta 2 power in all ROIs except ROI-1 [ROI-2: *F* (2, 34) = 4.96, *p* = 0.02, partial η^2^ = 0.23; ROI-3: *F* (2, 34) = 7.13, *p* < 0.01, partial η^2^ = 0.30; ROI-4: *F* (2, 34) = 5.40, *p* < 0.01, partial η^2^ = 0.24; and ROI-5: *F* (2, 34) = 5.59, *p* < 0.01, partial η^2^ = 0.25]. As shown in Fig 5, in the post-hoc test, the control design had a significantly greater absolute beta 2 power than the Japanese and tropical designs at ROI-2 and ROI-4 (ROI-2–Japanese: *p* = 0.04, tropical: *p* < 0.01; ROI-4–Japanese: *p* < 0.01, tropical: *p* = 0.02). In addition, in ROI-3 and ROI-5, the control design had significantly higher absolute beta 2 powers than the tropical design (ROI-3, *p* < 0.01; and ROI-5, *p* = 0.04).

**Fig 6:**
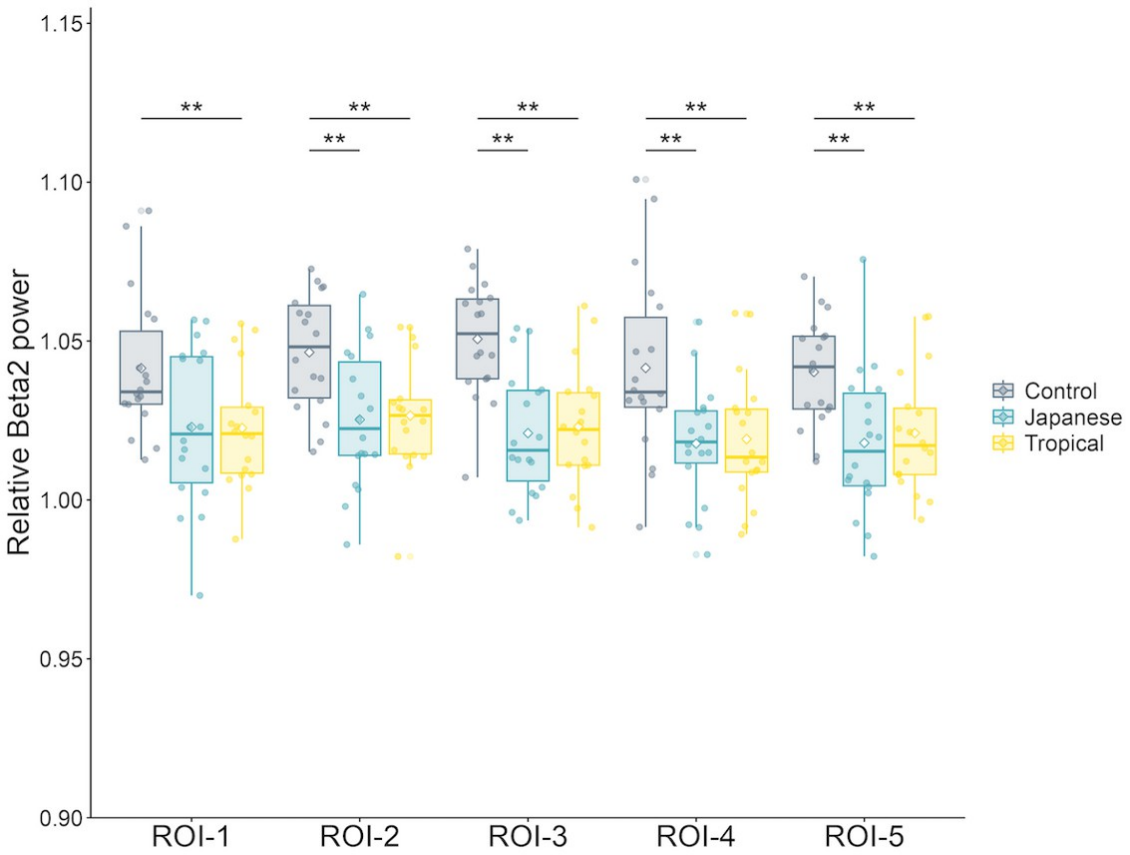
Comparison of the relative beta 2 power according to design in the 5-min exposure. The control design had significantly higher relative beta 2 power in ROI-2, ROI-3, ROI-4, and ROI-5 than the Japanese and tropical designs. The relative beta 2 power in ROI-1 was significantly higher in the control than in the tropical design (*p* < 0.01). Error bars are expressed as standard deviation. Asterisks above the bars indicate statistically significant between-design differences (**: *p* < 0.01). ROI, Region of interest; Control, control design; Japanese, Japanese design; Tropical, tropical design.

The design had significant effect on all ROIs in the 5-min exposure for relative beta 2 power [ROI-1, *F* (2, 34) = 7.19, *p* < 0.01, partial η^2^ = 0.30; ROI-2, *F* (2, 34) = 14.79, *p* < 0.01, partial η^2^ = 0.47; ROI-3, *F* (2, 34) = 31.45, *p* < 0.01, partial η^2^ = 0.65; ROI-4, *F* (2, 34) = 17.78, *p* < 0.01, partial η^2^ = 0.51; ROI-5, *F* (2, 34) = 28.35, *p* < 0.01, partial η^2^ = 0.63]. As depicted in Fig 6, in the post-hoc test, the control design had a significantly higher relative beta 2 power in ROI-2 and ROI-3 and ROI-4 and ROI-5 than the Japanese and tropical designs (ROI-2–Japanese: *p* < 0.01, tropical: *p* < 0.01; ROI-3–Japanese: *p* < 0.01, tropical: *p* < 0.01; ROI-4–Japanese: *p* < 0.01, tropical: *p* < 0.01; and ROI-5–Japanese: *p* < 0.01, tropical: *p* < 0.01). The control design had a significantly greater relative beta 2 power in ROI-1 than the tropical design (*p* < 0.01).

The relative beta 2 power of all ROIs in the Stroop task were significantly affected by the design [ROI-1: *F* (2, 34) = 4.40, *p* = 0.02, partial η^2^ = 0.21; ROI-2: *F* (2, 34) = 14.83, *p* < 0.01, partial η^2^ = 0.47; ROI-3: *F* (2, 34) = 20.95, *p* < 0.01, partial η^2^ = 0.56; ROI-4: *F* (2, 34) = 16.27, *p* < 0.01, partial η^2^ = 0.49; and ROI-5: *F* (2, 34) = 18.27, *p* < 0.01, partial η^2^ = 0.52].

**Fig 7:**
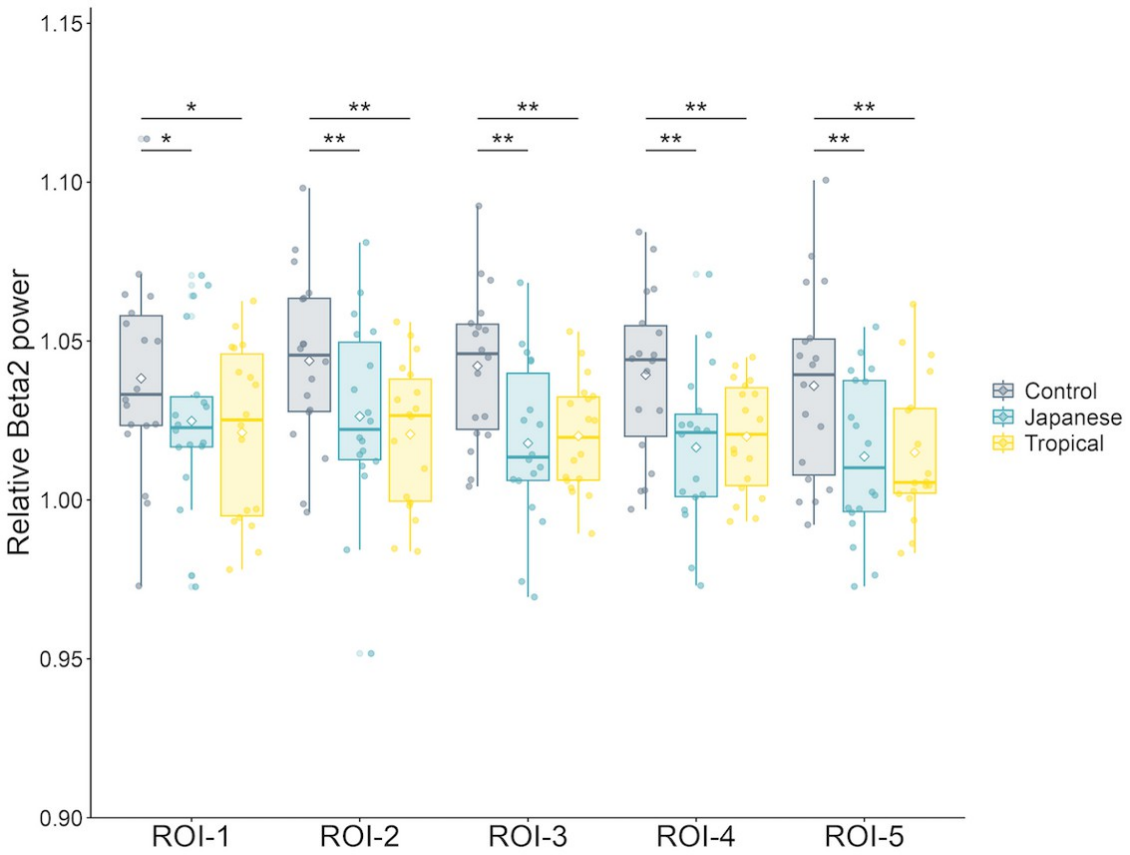
Comparison of the relative beta 2 power according to design in the Stroop task. All ROIs’ relative beta 2 power in the control design was significantly higher than that of the Japanese and tropical designs. Error bars are expressed as standard deviations. The asterisks above bars indicate statistically significant differences between designs (*: *p* < 0.05; **: *p* < 0.01). ROI, Region of interest; Control, control design; Japanese, Japanese design; Tropical, tropical design.

As presented in Fig 7, in the post-hoc test, compared with the Japanese and tropical designs, the control design had a significantly higher relative beta 2 power for all ROIs (ROI-1–Japanese: *p* = 0.046, tropical: *p* = 0.048; ROI-2–Japanese: *p* < 0.01, tropical: *p* < 0.01; ROI-3–Japanese: *p* < 0.01, tropical: *p* < 0.01; ROI-4–Japanese: *p* < 0.01, tropical: *p* < 0.01; and ROI-5–Japanese: *p* < 0.01, tropical: *p* < 0.01).

Significant main effect of design on relative beta 2 power was found for ROI-3 and ROI-4 in the 2-back task [ROI-3: *F* (2, 34) = 11.47, *p* < 0.01, partial η^2^ = 0.40; ROI-4: *F* (2, 34) = 7.48, *p* = 0.03, partial η^2^ = 0.31]. In the post-hoc test, the control design had a significantly higher relative beta 2 power for both ROIs (ROI-3–Japanese: *p* < 0.01, tropical: *p* < 0.01; ROI-4–Japanese: *p* = 0.03, tropical: *p* = 0.03) than the Japanese and tropical designs.

### Behavioral indices

The accuracy rate and reaction time in the Stroop task were analyzed using the two-way repeated measure ANOVA of design (control, Japanese, and tropical) × task condition (congruent, incongruent) (Table 3). Two-way repeated measure ANOVA using the accuracy rates of the Stroop task as dependent variables revealed that the task condition had a significant effect [*F* (1, 17) = 13.62, *p* < 0.01, partial η^2^ = 0.45]. Bonferroni’s multiple comparison test between task conditions indicated that the incongruent conditions had lower accuracy rates than the congruent conditions (*p* < 0.01). Two-way repeated measure ANOVA using the reaction time of the Stroop task as dependent variables revealed that the task condition had a significant effect [*F* (1, 17) = 73.06, *p* < 0.01, partial η^2^ = 0.81]. Bonferroni’s multiple comparison test between task conditions indicated that the incongruent conditions had a higher reaction time than the congruent conditions (*p* < 0.01). The interaction effects of the design (control, Japanese, and tropical) × task condition (congruent, incongruent) were insignificant for both the accuracy rate and reaction time. One-way repeated measure ANOVA showed no significant differences in accuracy rates and reaction time between designs (control, Japanese, and tropical) in the 1-back task and 2-back task.

**Table 3:** Accuracy rates and reaction time in the cognitive tasks at control, Japanese, and tropical designs.

**Accuracy (%)**	**Stroop_congruent**		×	**Stroop_incongruent**		**1-back task**		**2-back task**	
	Mean	SD		Mean	SD	Mean	SD	Mean	SD
**Control**	98.46	2.73		96.60	3.88	96.34	3.33	87.53	9.11
**Japanese**	99.54	1.07		97.53	2.31	96.93	3.28	87.44	11.15
**Tropical**	98.91	1.69		98.30	2.16	96.57	2.84	85.27	13.49
***F*-value**	Design factor: (2, 34) = 1.59					(2, 34) = 0.20		(2, 34) = 0.55	
	Task factor: (1, 17) = 13.62								
	Design × task condition: (2, 34) = 1.68								
**Partial η** ^**2**^	Design factor: 0.09					0.12		0.03	
	Task condition factor: 0.45								
	Design × task condition: 0.09								
***P*-value**	Design factor: 0.22					0.80		0.58	
	Task condition factor: ***< 0*.*01***								
	Design × task condition: 0.20								
**Post-hoc**	Task factor: ***congruent > incongruent (p < 0*.*01)***								
**RT (ms)**	**Stroop_congruent**		×	**Stroop_incongruent**		**1-back task**		**2-back task**	
	Mean	SD		Mean	SD	Mean	SD	Mean	SD
**Control**	555.89	76.45		625.96	117.43	648.02	118.64	1,103.09	303.31
**Japanese**	576.68	74.24		633.11	93.04	645.31	94.69	1,105.55	336.26
**Tropical**	557.11	60.74		610.97	78.96	646.68	107.29	1,117.95	318.26
***F*-value**	Design factor: (2, 34) = 1.05					(2, 34) = 0.01		(2, 34) = 0.04	
	Task condition factor: (1, 17) = 73.06								
	Design × task condition: (2, 34) = 0.76								
**partial η** ^**2**^	Design factor: 0.06					< 0.001		< 0.001	
	Task condition factor: 0.81								
	Design × task condition: 0.04								
***P*-value**	Design factor: 0.36					0.99		0.96	
	Task condition factor: ***< 0*.*01***								
	Design × task condition: 0.48								
**Post-hoc**	Task factor: ***congruent < incongruent (p < 0*.*01)***								

Values were presented as mean and standard deviation.

Bold and italic indicates statistically significant

Stroop task, Stroop color and word task; Stroop_congruent / incongruent, congruent / incongruent condition in Stroop task; 1-back, 1-back task; 2-back, 2-back task; Control, control design; Japanese, Japanese design; Tropical, tropical design; RT, reaction time; SD, standard deviation

## Discussion

### Psychological effects of the different design of greening (SDM and PANAS)

Two greening conditions were almost positively evaluated for space impression evaluated using the SDM. Unlike the tropical design, greening in the Japanese design was highly evaluated based on the “restless–calm” and “Western–Japanese” items. Therefore, space impression for the indoor environment improved with indoor greening. Further, the degree of calming with the Japanese design was larger than that with the tropical design. The degree of greenness could affect the viewer’s preference [32]. The psychological impression of ornamental foliage plants varies with the plants’ shape, size, distance to the estimator [33], and recognition of the plants [34]. Asaumi et al. [34] investigated the psychological effect of ornamental foliage plants in Japanese university students. Therefore, they evaluated ornamental foliage plants, popular and widely distributed in the Japanese market as restful. In this study, the green visibility rate and the distances to participants, but not the species and the shape and size of plants, were similar between the two designs (Fig 1 and Table 1). The species of ornamental foliage plants used in the tropical design were popular as ornamental foliage plants. Meanwhile, those used in Japanese design were used for outdoor facilities such as buildings and gardens. The elements used in the greening design that change the impression of indoor space remain controversial.

Each greening design’s positive and negative emotions differed after the 5-min exposure. No significant difference was observed between the greening design regarding positive affect (PA). The Japanese design had a lower NA than the control and tropical designs. Hence, greening with the Japanese design has decreased NA, improving emotional status. A meta-analysis [29] indicated that exposure to the natural environment has a medium to large effect in increasing positive and decreasing negative emotions. Grinde et al. [35] had evaluated approximately 50 empirical studies. Results showed that indoor plants had an impact on emotional status. Therefore, the emotion evoked by indoor space may differ from the greening design.

### Physiological and cognitive performance effects of different greening designs (HRV, EEG, and cognitive performance)

In this study, greening did not significantly affect the HRV. Hence, the autonomic nervous activity was not affected by greening. Yin et al. [13] showed that the indoor biophilic environment is associated with decreased blood pressure. Park et al. [14] revealed that the natural logarithm of LF/HF was significantly lower while performing a treatment task with foliage plants. Therefore, the sympathetic nervous system activity decreased with indoor greening or treatment with foliage plants. Conversely, Choi et al. [32] showed no changes in terms of HRV between interior spaces with different degrees of greening. McSweeney et al. [36] revealed that the participants rated the room with natural elements more positively than without. However, their HRV did not differ between with or without natural elements. This study’s HRV and psychological data results may support those of McSweeney et al.

The EEG results in 5-min exposure showed limited frequency bands and ROIs affected by the greening design. The tropical design had a significantly higher relative theta power in ROI-2 (right-frontal) than the control design. The control design had a significantly higher relative beta 2 power in ROI-2 (right-frontal), ROI-3 (left-posterior), ROI-4 (right-posterior), and ROI-5 (midline) than the Japanese and tropical designs. Further, the control design had a significantly higher relative beta 2 power in ROI-1 (left-frontal) than the tropical design. The relative theta reflects deep relaxation, unconsciousness, and optimal meditative state; in contrast, the relative beta 2 (relatively high beta) power reflects hyper-alertness and anxiety [27, 37]. Hence, the brain is relaxed or calm in the tropical design. Also, the Japanese and tropical designs suppress anxiety and tension in the brain. These calming effects and negative emotional ameliorating effects are based on SRT [11, 12] due to exposure to nature, which supports our hypothesis. Combined with the PANAS results, indoor greening with the tropical design promoted positive mood states, and indoor greening with the Japanese design suppressed negative mood states. Jeong and Park [18] showed that visual stimulation with real plants increased the relative theta power spectrum and decreased the relatively high beta (relative beta 2 in this study) power spectrum. Simultaneously, self-reporting using SDM and Profile of Mood State showed that participants had significantly higher “comfort,” “naturalness,” and “relaxation” scores and increased positive mood states when viewing real plants [20]. Chen, He, and Yu [19] found that theta power was higher during restorative environmental experiences in forest gardens than during nonrestorative experiences and that there was a strong negative association between theta oscillations and subjective fatigue. The current study’s findings were consistent with those of the previous studies [13, 38].

There were no significant differences between designs regarding the performance of cognitive tasks (Stroop, 1-back, and 2-back tasks) performed after 5 min of exposure in each greening design. In a previous study by Yin et al. [13], performance in the visual backward digital span task was higher in green spaces (biophilic) than in nongreen spaces (nonbiophilic). Therefore, indoor greening is associated with short-term memory. These results differed from those of the current study. This may be attributed to the effect of differences in indoor greening. Furthermore, the effect of indoor greening may vary based on the cognitive task (cognitive function). Yin et al. [13] found that greening did not affect reaction time and Stroop task performance.

During the cognitive tasks, the control design had a significantly higher relative beta 2 (relatively high beta) power for all ROIs than the Japanese and tropical designs in the Stroop task. The frontal theta power during cognitive tasks indicates cognitive workload (Chikhi et al. [28], meta-analysis). Beta 2 involves top–down information processes [39, 40]. Pavlov and Kotchoubey [41] reported that a high cognitive task load increases beta 2 activity. Contrary to the hypothesis based on ART theory, no differences in performance on cognitive tasks were found between designs in the present study [9, 10]. However, the control design had a significantly higher relative beta 2 in the whole brain than the Japanese or tropical designs. In addition, the control design had a significantly higher relative beta 2 power in ROI-3 (left-posterior) and ROI-4 (right-posterior) in the 2-back task. This phenomenon could explain the neural efficiency hypothesis, which states that a higher neural activation in equivalent performance indicates less efficient neural processing [42]. Cognitive tasks in rooms without green plants may reflect a greater increase in neural resources and a lower efficiency in neural processing. In contrast, the results suggest that rooms with green plants had neural efficiency in responding to cognitive tasks with fewer neural resources. Therefore, room greening increases neural efficiency during cognitive tasks. Recently, Rhee et al. [38] recorded the participants’ EEG during cognitive tasks and showed the indoor environment with nature had restorative and cognitive benefits. Zhang et al. [16] have hypothesized the “effortless” processing of the brain in the natural environment. Furthermore, this study can help evaluate the effort required for cognitive tasks in biophilic designs using multichannel EEG. It may explain the effect of greening on cognitive work efficiency.

### Limitations

The current study evaluated the physiological and psychological effects of the Japanese and tropical designs using EEG, HRV, SDM, and PANAS. Therefore, future studies should include EEG data and subjective evaluation results for various biophilic designs. The EEG data during the cognitive tasks used in this study showed that the neural efficiency increased in the greening room. However, in contrast to previous studies, the greening room did not affect behavioral performance, which was evaluated using parameters such as reaction time and accuracy rate. Nevertheless, further studies should examine the impact of different biophilic designs on cognitive tasks and the combination of cognitive tasks affected by greening.

Participants’ understanding of the research purpose through the instructions for informed consent may unconsciously affect the results of subjective and behavioral indicators. So, the blinded study design will be administered in future research.

## Conclusion

The self-reported data on emotions (SDM, PANAS) showed that the tropical design promoted positive mood states, and the Japanese design inhibited negative mood states. These self-reported data were supported by the results of EEG. Furthermore, indoor greening enhanced neural efficiency during cognitive tasks. The findings can provide direct evidence for improvement of the office environment and health status of workers. Moreover, they can help to understand the effects of different biophilic designs on people’s mental state and cognitive performance.

## Supporting information

S1 Table The mean scores obtained via the evaluation using SDM for all spaces. (DOCX)

S2 Table Summary of the analysis of variance on HRV in 5-min exposure and cognitive tasks. (DOCX)

S3 Table Summary of the analysis of variance results on EEG theta powers in the 5-min exposure, Stroop, 1-back, and 2-back tasks. (DOCX)

S4 Table Summary of the analysis of variance results on EEG alpha 1 powers in the 5-min exposure, Stroop, 1-back, and 2-back tasks. (DOCX)

S5 Table Summary of the analysis of variance results on EEG alpha 2 powers in the 5-min exposure, Stroop, 1-back, and 2-back tasks. (DOCX)

S6 Table Summary of the analysis of variance results on EEG beta 1 powers in the 5-min exposure, Stroop, 1-back, and 2-back tasks. (DOCX)

S7 Table Summary of the analysis of variance results on EEG beta 2 powers in the 5-min exposure, Stroop, 1-back, and 2-back tasks. (DOCX)
