# Supplementary material for: The effects of different designs of indoor biophilic greening on psychological and physiological responses and cognitive performance of office workers
Source: PLoS One. 2024 Jul 26;19(7):e0307934. doi: 10.1371/journal.pone.0307934 (PMC11280145; doi:10.1371/journal.pone.0307934)
Supplement: S1 Table — (DOCX) [file pone.0307934.s001.docx]

**S1 Table. The mean scores of the evaluation performed using the SDM for all spaces.**

|  | Uncomfortable–comfortable | | | Restless–calm | |  | Quiet–noisy | |  | Unfamiliar–familiar | | | Dark–blight | |  |
| --- | --- | --- | --- | --- | --- | --- | --- | --- | --- | --- | --- | --- | --- | --- | --- |
|  | Mean | SD |  | Mean | SD |  | Mean | SD |  | Mean | SD |  | Mean | SD |  |
| Control | 3.61 | 1.42 |  | 3.56 | 1.43 |  | 1.89 | 0.83 |  | 3.17 | 1.58 |  | 3.72 | 1.63 |  |
| Japanese | 5.72 | 0.67 |  | 6.00 | 0.77 |  | 3.56 | 1.72 |  | 5.61 | 0.70 |  | 5.17 | 1.15 |  |
| Tropical | 5.11 | 0.58 |  | 4.50 | 1.30 |  | 4.17 | 1.76 |  | 5.00 | 0.60 |  | 5.33 | 1.10 |  |
| *F-*value | (2, 34) = 23.41 | | | (2, 34) = 19.39 | |  | (2, 34) = 12.77 | |  | (2, 34) = 24.11 | |  | (2, 34) = 6.57 | |  |
| Partial η^2^ | 0.58 |  |  | 0.76 |  |  | 0.43 |  |  | 0.59 |  |  | 0.28 |  |  |
| *P*-value | ***< 0.01*** |  |  | ***< 0.01*** |  |  | ***< 0.01*** |  |  | ***< 0.01*** |  |  | ***< 0.01*** |  |  |
| Post-hoc | ***Control < Japanese, Tropical;***  ***Tropical < Japanese*** | | | ***Control < Japanese;***  ***Tropical < Japanese*** | | | ***Control < Japanese, Tropical*** | | | ***Control < Japanese, Tropical;***  ***Tropical < Japanese*** | | | ***Control < Japanese, Tropical*** | | |
|  |  |  |  |  |  |  |  |  |  |  |  |  |  |  |  |
|  | Tightness–spacious | | | Dislike–like | |  | Western–Japanese style | | | Not healed–healed | | | Artificial–natural | | |
|  | Mean | SD |  | Mean | SD |  | Mean | SD |  | Mean | SD |  | Mean | SD |  |
| Control | 3.28 | 1.32 |  | 3.00 | 1.19 |  | 3.33 | 0.33 |  | 2.17 | 1.10 |  | 1.44 | 0.62 |  |
| Japanese | 5.39 | 0.92 |  | 5.94 | 0.87 |  | 4.56 | 1.76 |  | 5.94 | 0.80 |  | 5.94 | 0.80 |  |
| Tropical | 4.33 | 0.84 |  | 5.33 | 1.09 |  | 2.50 | 0.99 |  | 5.06 | 0.80 |  | 5.39 | 0.78 |  |
| *F-*value | (2, 34) = 17.69 | |  | (2, 34) = 34.31 | |  | (2, 34) = 13.39 | |  | (2, 34) = 69.77 | |  | (2, 34) = 205.09 | | |
| Partial η^2^ | 0.05 |  |  | 0.67 |  |  | 0.44 |  |  | 0.80 |  |  | 0.92 |  |  |
| *P*-value | ***< 0.01*** |  |  | ***< 0.01*** |  |  | ***< 0.01*** |  |  | ***< 0.01*** |  |  | ***< 0.01*** |  |  |
| post-hoc | ***Control < Japanese;***  ***Tropical < Japanese*** | | | ***Control < Japanese, Tropical*** | | | ***Control, Japanese > Tropical*** | | | ***Control < Japanese, Tropical;***  ***Tropical < Japanese*** | | | ***Control < Japanese, Tropical*** | | |

Bold and italic - indicates statistically significant

Control, control design; Japanese, Japanese design; Tropical, tropical design; SD, standard deviation
