# Supplementary material for: The effects of different designs of indoor biophilic greening on psychological and physiological responses and cognitive performance of office workers
Source: PLoS One. 2024 Jul 26;19(7):e0307934. doi: 10.1371/journal.pone.0307934 (PMC11280145; doi:10.1371/journal.pone.0307934)
Supplement: S2 Table — (DOCX) [file pone.0307934.s002.docx]

**S2 Table. Summary of the analysis of variance on HRV in 5-min exposure and cognitive tasks.**

| **lnHF** | Control | | |  | | Japanese | | | |  | | Tropical | | | |
| --- | --- | --- | --- | --- | --- | --- | --- | --- | --- | --- | --- | --- | --- | --- | --- |
|  | Mean | SD | |  | | Mean | | SD | |  | | Mean | | SD | |
| 5-min exposure | 0.174 | 0.521 | |  | | 0.113 | | 0.568 | |  | | 0.140 | | 0.487 | |
| Cognitive task | 0.0917 | 0.573 | |  | | 0.109 | | 0.521 | |  | | 0.0083 | | 0.553 | |
| *F* value | Design, *F* (2, 34) = 1.02  Task, *F* (1,17) = 2.48  Design*Task interaction, *F* (2.34) = 2.15 | | | | | | | | | | | | | | |
| Partial η^2^ | Design, 0.056  Task, 0.127  Design*Task interaction, 0.112 | | | | | | | | | | | | | | |
| *p* value | Design, 0.372  Task, 0.133  Design*Task interaction, 0.132 | | | | | | | | | | | | | | |
| Post-hoc | none | | | | | | | | | | | | | | |
| **LF/HF** | Control | | | |  | | Japanese | | | |  | | Tropical | | |
|  | Mean | | SD | |  | | Mean | | SD | |  | | Mean | | SD |
| 5-min exposure | 0.174 | | 0.521 | |  | | 0.113 | | 0.568 | |  | | 0.140 | | 0.487 |
| Cognitive task | 0.0917 | | 0.573 | |  | | 0.109 | | 0.521 | |  | | 0.0083 | | 0.553 |
| *F* value | Design, *F* (2, 34) = 0.115  Task, *F* (1,17) = 8.879  Design*Task interaction, *F* (2.34) = 0.623 | | | | | | | | | | | | | | |
| Partial η^2^ | Design, 0.007  Task, 0.341  Design*Task interaction, 0.035 | | | | | | | | | | | | | | |
| *p* value | Design, 0.892  Task, 0.009  Design*Task interaction, 0.542 | | | | | | | | | | | | | | |
| Post-hoc | Task, **5-min > Cognitive task** (*p* = 0.009) | | | | | | | | | | | | | | |
| **HR** | Control | | | |  | | Japanese | | | |  | | Tropical | | |
|  | Mean | | SD | |  | | Mean | | SD | |  | | Mean | | SD |
| 5-min exposure | 77.7 | | 9.63 | |  | | 77.9 | | 10.8 | |  | | 77.6 | | 8.69 |
| Cognitive task | 79.6 | | 9.64 | |  | | 79.6 | | 10.2 | |  | | 79.9 | | 9.59 |
| *F* value | Design, *F* (2, 34) = 0.002  Task, *F* (1,17) = 5.459  Design*Task interaction, *F* (2.34) = 0.359 | | | | | | | | | | | | | | |
| Partial η^2^ | Design, 0.000  Task, 0.243  Design*Task interaction, 0.021 | | | | | | | | | | | | | | |
| *p* value | Design, 0.998  Task, 0.032  Design*Task interaction, 0.701 | | | | | | | | | | | | | | |
| Post-hoc | Task, **5-min < Cognitive task** (*p* = 0.032) | | | | | | | | | | | | | | |
