# Supplementary material for: The effects of different designs of indoor biophilic greening on psychological and physiological responses and cognitive performance of office workers
Source: PLoS One. 2024 Jul 26;19(7):e0307934. doi: 10.1371/journal.pone.0307934 (PMC11280145; doi:10.1371/journal.pone.0307934)
Supplement: S3 Table — (DOCX) [file pone.0307934.s003.docx]

**S3 Table. Summary of the analysis of variance results on EEG theta powers in the 5-min exposure, Stroop, 1-back, and 2-back tasks.**

| **EEG theta_Absolute power** | |  |  |  |  |  |  |  |  |  |  |  |  |  |
| --- | --- | --- | --- | --- | --- | --- | --- | --- | --- | --- | --- | --- | --- | --- |
| **Exposure** | ROI-1 | |  | ROI-2 | |  | ROI-3 | |  | ROI-4 | |  | ROI-5 | |
|  | Mean | SD |  | Mean | SD |  | Mean | SD |  | Mean | SD |  | Mean | SD |
| Control | 31.68 | 1.88 |  | 32.65 | 2.11 |  | 34.53 | 2.07 |  | 35.02 | 2.18 |  | 35.02 | 2.31 |
| Japanese | 31.51 | 1.69 |  | 32.14 | 2.06 |  | 34.55 | 2.50 |  | 34.83 | 2.32 |  | 34.90 | 1.97 |
| Tropical | 31.50 | 2.09 |  | 32.35 | 2.59 |  | 34.28 | 2.30 |  | 34.6- | 2.43 |  | 34.60 | 2.62 |
| *F*-value | (2, 34) = 0.11 |  |  | (2, 34) = 0.51 |  |  | (2, 34) = 0.36 |  |  | (2, 34) = 0.46 |  |  | (2, 34) = 0.74 |  |
| Partial η^2^ | < 0.01 |  |  | 0.03 |  |  | 0.02 |  |  | 0.05 |  |  | 0.04 |  |
| *P*-value | 0.90 |  |  | 0.60 |  |  | 0.70 |  |  | 0.66 |  |  | 0.49 |  |
| Post-hoc | - |  |  | - |  |  | - |  |  | - |  |  | - |  |
| **Stroop task** | ROI-1 | |  | ROI-2 | |  | ROI-3 | |  | ROI-4 | |  | ROI-5 | |
|  | Mean | SD |  | Mean | SD |  | Mean | SD |  | Mean | SD |  | Mean | SD |
| Control | 32.20 | 0.02 |  | 32.07 | 1.89 |  | 33.68 | 2.64 |  | 34.39 | 2.61 |  | 33.87 | 2.62 |
| Japanese | 32.68 | 3.80 |  | 32.50 | 2.94 |  | 33.76 | 3.15 |  | 34.08 | 3.11 |  | 34.36 | 2.75 |
| Tropical | 34.00 | 4.59 |  | 34.59 | 5.08 |  | 34.40 | 3.39 |  | 35.09 | 3.53 |  | 35.02 | 4.58 |
| *F*-value | (2, 34) = 2.11 |  |  | (2, 34) = 3.87 |  |  | (2, 34) = 0.98 |  |  | (2, 34) = 1.86 |  |  | (2, 34) = 1.12 |  |
| Partial η^2^ | 0.11 |  |  | 0.19 |  |  | 0.06 |  |  | 0.10 |  |  | 0.06 |  |
| *P*-value | 0.14 |  |  | ***0.03*** |  |  | 0.39 |  |  | 0.17 |  |  | 0.34 |  |
| Post-hoc | - |  |  | n.s. |  |  | - |  |  | - |  |  | - |  |
| **1-back** | ROI-1 | |  | ROI-2 | |  | ROI-3 | |  | ROI-4 | |  | ROI-5 | |
|  | Mean | SD |  | Mean | SD |  | Mean | SD |  | Mean | SD |  | Mean | SD |
| Control | 34.69 | 3.74 |  | 35.53 | 3.78 |  | 37.08 | 3.68 |  | 38.45 | 4.02 |  | 36.21 | 3.80 |
| Japanese | 33.87 | 3.79 |  | 34.42 | 4.16 |  | 36.25 | 4.30 |  | 37.28 | 4.29 |  | 35.48 | 3.64 |
| Tropical | 33.82 | 3.14 |  | 34.56 | 3.59 |  | 36.06 | 3.73 |  | 37.14 | 3.62 |  | 35.39 | 3.75 |
| *F*-value | (2, 34) = 1.94 |  |  | (2, 34) = 2.81 |  |  | (2, 34) = 2.55 |  |  | (2, 34) = 2.80 |  |  | (2, 34) = 1.84 |  |
| Partial η^2^ | 0.11 |  |  | 0.15 |  |  | 0.14 |  |  | 0.15 |  |  | 0.10 |  |
| *P*-value | 0.16 |  |  | 0.08 |  |  | 0.09 |  |  | 0.08 |  |  | 0.18 |  |
| Post-hoc | - |  |  | - |  |  | - |  |  | - |  |  | - |  |
| **2-back** | ROI-1 | |  | ROI-2 | |  | ROI-3 | |  | ROI-4 | |  | ROI-5 | |
|  | Mean | SD |  | Mean | SD |  | Mean | SD |  | Mean | SD |  | Mean | SD |
| Control | 31.84 | 3.72 |  | 31.82 | 3.31 |  | 31.75 | 4.19 |  | 32.16 | 3.17 |  | 32.35 | 3.93 |
| Japanese | 31.61 | 3.51 |  | 31.77 | 3.23 |  | 31.75 | 3.75 |  | 31.53 | 3.36 |  | 32.14 | 3.36 |
| Tropical | 30.96 | 7.87 |  | 30.79 | 7.82 |  | 30.53 | 7.80 |  | 31.35 | 7.93 |  | 31.72 | 8.22 |
| *F*-value | (2, 34) = 0.13 |  |  | (2, 34) = 0.23 |  |  | (2, 34) = 0.37 |  |  | (2, 34) = 0.14 |  |  | (2, 34) = 0.07 |  |
| Partial η^2^ | 0.01 |  |  | 0.01 |  |  | 0.02 |  |  | 0.01 |  |  | 0.00 |  |
| *P*-value | 0.88 |  |  | 0.80 |  |  | 0.69 |  |  | 0.87 |  |  | 0.94 |  |
| Post-hoc | - |  |  | - |  |  | - |  |  | - |  |  | - |  |
|  |  |  |  |  |  |  |  |  |  |  |  |  |  |  |
| **EEG theta_Relative power** | |  |  |  |  |  |  |  |  |  |  |  |  |  |
| **Exposure** | ROI-1 | |  | ROI-2 | |  | ROI-3 | |  | ROI-4 | |  | ROI-5 | |
|  | Mean | SD |  | Mean | SD |  | Mean | SD |  | Mean | SD |  | Mean | SD |
| Control | 1.24 | 0.05 |  | 1.23 | 0.05 |  | 1.20 | 0.06 |  | 1.20 | 0.05 |  | 1.25 | 0.04 |
| Japanese | 1.24 | 0.05 |  | 1.24 | 0.05 |  | 1.21 | 0.05 |  | 0.21 | 0.05 |  | 1.25 | 0.03 |
| Tropical | 1.27 | 0.04 |  | 1.26 | 0.05 |  | 1.22 | 0.04 |  | 1.22 | 0.05 |  | 1.26 | 0.04 |
| *F*-value | (2, 34) = 2.98 |  |  | (2, 34) = 0.58 |  |  | (2, 34) = 1.79 |  |  | (2, 34) = 0.89 |  |  | (2, 34) = 0.71 |  |
| Partial η^2^ | 0.15 |  |  | 0.26 |  |  | 0.10 |  |  | 0.05 |  |  | 0.04 |  |
| *P*-value | 0.06 |  |  | ***< 0.01*** |  |  | 0.18 |  |  | 0.42 |  |  | 0.50 |  |
| Post-hoc | - |  |  | ***Control < Tropical*** | |  | - |  |  | - |  |  | - |  |
| **Stroop task** | ROI-1 | |  | ROI-2 | |  | ROI-3 | |  | ROI-4 | |  | ROI-5 | |
|  | Mean | SD |  | Mean | SD |  | Mean | SD |  | Mean | SD |  | Mean | SD |
| Control | 1.24 | 0.09 |  | 1.22 | 0.05 |  | 1.21 | 0.07 |  | 1.20 | 0.07 |  | 1.27 | 0.11 |
| Japanese | 1.21 | 0.06 |  | 1.22 | 0.06 |  | 1.20 | 0.05 |  | 1.20 | 0.06 |  | 1.25 | 0.05 |
| Tropical | 1.23 | 0.07 |  | 1.23 | 0.07 |  | 1.21 | 0.06 |  | 1.20 | 0.06 |  | 1.26 | 0.06 |
| *F*-value | (2, 34) = 0.67 |  |  | (2, 34) = 0.39 |  |  | (2, 34) = 0.52 |  |  | (2, 34) = 0.12 |  |  | (2, 34) = 0.46 |  |
| Partial η^2^ | 0.04 |  |  | 0.02 |  |  | 0.03 |  |  | 0.01 |  |  | 0.03 |  |
| *P*-value | 0.52 |  |  | 0.68 |  |  | 0.60 |  |  | 0.86 |  |  | 0.63 |  |
| Post-hoc | - |  |  | - |  |  | - |  |  | - |  |  | - |  |
| **1-back** | ROI-1 | |  | ROI-2 | |  | ROI-3 | |  | ROI-4 | |  | ROI-5 | |
|  | Mean | SD |  | Mean | SD |  | Mean | SD |  | Mean | SD |  | Mean | SD |
| Control | 1.20 | 0.07 |  | 1.20 | 0.08 |  | 1.19 | 0.07 |  | 1.20 | 0.06 |  | 1.24 | 0.06 |
| Japanese | 1.22 | 0.10 |  | 1.22 | 0.09 |  | 1.23 | 0.11 |  | 1.20 | 0.09 |  | 1.25 | 0.08 |
| Tropical | 1.20 | 0.06 |  | 1.20 | 0.05 |  | 1.18 | 0.04 |  | 1.19 | 0.04 |  | 1.23 | 0.05 |
| *F*-value | (2, 34) = 0.48 |  |  | (2, 34) = 0.54 |  |  | (2, 34) = 2.24 |  |  | (2, 34) = 0.08 |  |  | (2, 34) = 0.84 |  |
| Partial η^2^ | 0.03 |  |  | 0.03 |  |  | 0.12 |  |  | 0.01 |  |  | 0.05 |  |
| *P*-value | 0.62 |  |  | 0.59 |  |  | 0.12 |  |  | 0.92 |  |  | 0.44 |  |
| Post-hoc | - |  |  | - |  |  | - |  |  | - |  |  | - |  |
| **2-back** | ROI-1 | |  | ROI-2 | |  | ROI-3 | |  | ROI-4 | |  | ROI-5 | |
|  | Mean | SD |  | Mean | SD |  | Mean | SD |  | Mean | SD |  | Mean | SD |
| Control | 1.21 | 0.07 |  | 1.22 | 0.07 |  | 1.21 | 0.08 |  | 1.21 |  |  | 1.25 |  |
| Japanese | 1.21 | 0.05 |  | 1.22 | 0.05 |  | 1.21 | 0.06 |  | 1.20 |  |  | 1.25 |  |
| Tropical | 1.22 | 0.06 |  | 1.22 | 0.05 |  | 1.20 | 0.06 |  | 1.19 |  |  | 1.26 |  |
| *F*-value | (2, 34) = 0.47 |  |  | (2, 34) = 0.03 |  |  | (2, 34) = 3.40 |  |  | (2, 34) = 0.63 |  |  | (2, 34) = 0.34 |  |
| Partial η^2^ | 0.03 |  |  | 0.01 |  |  | 0.02 |  |  | 0.04 |  |  | 0.02 |  |
| *P*-value | 0.63 |  |  | 0.96 |  |  | 0.71 |  |  | 0.54 |  |  | 0.68 |  |
| Post-hoc | - |  |  | - |  |  | - |  |  | - |  |  | - |  |

EEG signals data from 32 sites were arranged into the five regions of interests (ROIs). ROI-1, left-frontal (FP1, F3, F7); ROI-2, right-frontal (FP2, F4, F8); ROI-3, left-posterior (P3, P7, O1); ROI-4, right-posterior (P4, P8, O2); ROI-5, midline (Fz, Cz, Pz).

Bold and italic - indicates statistically significant

Exposure, 5-min exposure**;** Stroop task, stroop color and word task; 1-back, 1-back task; 2-back, 2-back task; Control, control design; Japanese, Japanese design; Tropical, tropical design; SD, standard deviation; n.s., not significant
