# Supplementary material for: The effects of different designs of indoor biophilic greening on psychological and physiological responses and cognitive performance of office workers
Source: PLoS One. 2024 Jul 26;19(7):e0307934. doi: 10.1371/journal.pone.0307934 (PMC11280145; doi:10.1371/journal.pone.0307934)
Supplement: S4 Table — (DOCX) [file pone.0307934.s004.docx]

**S4 Table. Summary of the analysis of variance results on EEG alpha 1 powers in the 5-min exposure, Stroop, 1-back, and 2-back tasks.**

| **EEG alpha 1_Absolute power** | |  |  |  |  |  |  |  |  |  |  |  |  |  |
| --- | --- | --- | --- | --- | --- | --- | --- | --- | --- | --- | --- | --- | --- | --- |
| **Exposure** | ROI-1 | |  | ROI-2 | |  | ROI-3 | |  | ROI-4 | |  | ROI-5 | |
|  | Mean | SD |  | Mean | SD |  | Mean | SD |  | Mean | SD |  | Mean | SD |
| Control | 33.87 | 3.90 |  | 34.16 | 4.01 |  | 37.14 | 4.42 |  | 37.98 | 4.81 |  | 36.79 | 4.24 |
| Japanese | 33.00 | 3.22 |  | 33.24 | 3.55 |  | 36.30 | 4.26 |  | 36.99 | 4.08 |  | 36.10 | 3.58 |
| Tropical | 32.63 | 3.29 |  | 32.98 | 3.62 |  | 35.70 | 3.86 |  | 36.39 | 3.68 |  | 35.53 | 3.94 |
| *F*-value | (2, 34) = 2.32 |  |  | (2, 34) = 1.86 |  |  | (2, 34) = 3.06 |  |  | (2, 34) = 2.67 |  |  | (2, 34) = 3.04 |  |
| Partial η^2^ | 0.12 |  |  | 0.10 |  |  | 0.15 |  |  | 0.14 |  |  | 0.15 |  |
| *P*-value | 0.11 |  |  | 0.17 |  |  | 0.06 |  |  | 0.08 |  |  | 0.06 |  |
| Post-hoc | - |  |  | - |  |  | - |  |  | - |  |  | - |  |
| **Stroop task** | ROI-1 | |  | ROI-2 | |  | ROI-3 | |  | ROI-4 | |  | ROI-5 | |
|  | Mean | SD |  | Mean | SD |  | Mean | SD |  | Mean | SD |  | Mean | SD |
| Control | 32.89 | 4.27 |  | 32.60 | 3.60 |  | 34.92 | 4.75 |  | 36.00 | 5.07 |  | 34.50 | 4.81 |
| Japanese | 33.00 | 4.61 |  | 32.64 | 4.19 |  | 34.49 | 4.91 |  | 35.01 | 5.12 |  | 34.54 | 4.19 |
| Tropical | 34.11 | 4.06 |  | 34.58 | 4.41 |  | 35.24 | 4.71 |  | 36.08 | 4.49 |  | 35.21 | 4.82 |
| *F*-value | (2, 34) = 2.10 |  |  | (2, 34) = 5.96 |  |  | (2, 34) = 0.61 |  |  | (2, 34) = 1.42 |  |  | (2, 34) = 0.684 |  |
| Partial η^2^ | 0.11 |  |  | 0.26 |  |  | 0.04 |  |  | 0.08 |  |  | 0.04 |  |
| *P*-value | 0.14 |  |  | ***< 0.01*** |  |  | 0.55 |  |  | 0.26 |  |  | 0.51 |  |
| Post-hoc | - |  |  | ***Control < Tropical*** | |  | - |  |  | - |  |  | - |  |
| **1-back** | ROI-1 | |  | ROI-2 | |  | ROI-3 | |  | ROI-4 | |  | ROI-5 | |
|  | Mean | SD |  | Mean | SD |  | Mean | SD |  | Mean | SD |  | Mean | SD |
| Control | 31.96 | 4.38 |  | 32.51 | 4.46 |  | 33.00 | 4.61 |  | 33.72 | 5.38 |  | 32.92 | 5.03 |
| Japanese | 32.58 | 4.56 |  | 33.18 | 4.01 |  | 32.82 | 5.06 |  | 34.18 | 4.54 |  | 33.38 | 4.06 |
| Tropical | 31.68 | 4.02 |  | 32.15 | 4.03 |  | 33.06 | 4.22 |  | 32.71 | 5.07 |  | 32.96 | 4.37 |
| *F*-value | (2, 34) = 0.53 |  |  | (2, 34) = 1.10 |  |  | (2, 34) = 0.40 |  |  | (2, 34) = 1.61 |  |  | (2, 34) = 0.27 |  |
| Partial η^2^ | 0.03 |  |  | 0.06 |  |  | 0.00 |  |  | 0.09 |  |  | 0.02 |  |
| *P*-value | 0.59 |  |  | 0.35 |  |  | 0.96 |  |  | 0.22 |  |  | 0.77 |  |
| Post-hoc | - |  |  | - |  |  | - |  |  | - |  |  | - |  |
| **2-back** | ROI-1 | |  | ROI-2 | |  | ROI-3 | |  | ROI-4 | |  | ROI-5 | |
|  | Mean | SD |  | Mean | SD |  | Mean | SD |  | Mean | SD |  | Mean | SD |
| Control | 31.09 | 4.18 |  | 30.91 | 4.02 |  | 31.23 | 5.05 |  | 31.82 | 4.52 |  | 31.55 | 4.34 |
| Japanese | 30.59 | 3.81 |  | 30.62 | 3.66 |  | 31.09 | 4.71 |  | 31.40 | 4.28 |  | 31.17 | 4.10 |
| Tropical | 32.11 | 3.48 |  | 31.83 | 3.60 |  | 32.16 | 4.13 |  | 33.15 | 4.10 |  | 32.79 | 4.18 |
| *F*-value | (2, 34) = 2.23 |  |  | (2, 34) = 1.14 |  |  | (2, 34) = 1.10 |  |  | (2, 34) = 2.66 |  |  | (2, 34) = 4.48 |  |
| Partial η^2^ | 0.12 |  |  | 0.06 |  |  | 0.06 |  |  | 0.14 |  |  | 0.21 |  |
| *P*-value | 0.12 |  |  | 0.33 |  |  | 0.35 |  |  | 0.08 |  |  | ***0.02*** |  |
| Post-hoc | - |  |  | - |  |  | - |  |  | - |  |  | ***Japanese < Tropical*** | |
|  |  |  |  |  |  |  |  |  |  |  |  |  |  |  |
| **EEG alpha 1_Relative power** | |  |  |  |  |  |  |  |  |  |  |  |  |  |
| **Exposure** | ROI-1 | |  | ROI-2 | |  | ROI-3 | |  | ROI-4 | |  | ROI-5 | |
|  | Mean | SD |  | Mean | SD |  | Mean | SD |  | Mean | SD |  | Mean | SD |
| Control | 1.32 | 0.12 |  | 1.29 | 0.10 |  | 1.29 | 0.11 |  | 1.31 | 0.13 |  | 1.31 | 0.10 |
| Japanese | 1.30 | 0.12 |  | 1.29 | 0.11 |  | 1.27 | 0.92 |  | 1.29 | 0.08 |  | 1.30 | 0.08 |
| Tropical | 1.31 | 0.09 |  | 1.29 | 0.08 |  | 1.27 | 0.08 |  | 1.28 | 0.08 |  | 1.29 | 0.06 |
| *F*-value | (2, 34) = 0.51 |  |  | (2, 34) = 0.02 |  |  | (2, 34) = 1.00 |  |  | (2, 34) = 1.33 |  |  | (2, 34) = 1.22 |  |
| Partial η^2^ | 0.03 |  |  | 0.00 |  |  | 0.06 |  |  | 0.07 |  |  | 0.07 |  |
| *P*-value | 0.60 |  |  | 0.98 |  |  | 0.38 |  |  | 0.28 |  |  | 0.31 |  |
| Post-hoc | - |  |  | - |  |  | - |  |  | - |  |  | - |  |
| **Stroop task** | ROI-1 | |  | ROI-2 | |  | ROI-3 | |  | ROI-4 | |  | ROI-5 | |
|  | Mean | SD |  | Mean | SD |  | Mean | SD |  | Mean | SD |  | Mean | SD |
| Control | 1.26 | 0.09 |  | 1.24 | 0.09 |  | 1.25 | 0.09 |  | 1.25 | 0.10 |  | 1.28 | 0.09 |
| Japanese | 1.22 | 0.10 |  | 1.22 | 0.08 |  | 1.22 | 0.10 |  | 1.22 | 0.10 |  | 1.25 | 0.09 |
| Tropical | 1.23 | 0.09 |  | 1.24 | 0.08 |  | 1.23 | 0.09 |  | 1.23 | 0.08 |  | 1.26 | 0.08 |
| *F*-value | (2, 34) = 1.77 |  |  | (2, 34) = 0.68 |  |  | (2, 34) = 1.03 |  |  | (2, 34) = 0.99 |  |  | (2, 34) = 2.33 |  |
| Partial η^2^ | 0.09 |  |  | 0.04 |  |  | 0.06 |  |  | 0.06 |  |  | 0.12 |  |
| *P*-value | 0.19 |  |  | 0.51 |  |  | 0.37 |  |  | 0.38 |  |  | 0.11 |  |
| Post-hoc | - |  |  | - |  |  | - |  |  | - |  |  | - |  |
| **1-back** | ROI-1 | |  | ROI-2 | |  | ROI-3 | |  | ROI-4 | |  | ROI-5 | |
|  | Mean | SD |  | Mean | SD |  | Mean | SD |  | Mean | SD |  | Mean | SD |
| Control | 1.19 | 0.11 |  | 1.19 | 0.10 |  | 1.20 | 0.10 |  | 1.21 | 0.11 |  | 1.23 | 0.09 |
| Japanese | 1.19 | 0.07 |  | 1.19 | 0.06 |  | 1.21 | 0.07 |  | 1.21 | 0.07 |  | 1.22 | 0.06 |
| Tropical | 1.20 | 0.08 |  | 1.19 | 0.07 |  | 1.19 | 0.07 |  | 1.20 | 0.07 |  | 1.21 | 0.07 |
| *F*-value | (2, 34) = 0.20 |  |  | (2, 34) = 0.08 |  |  | (2, 34) = 0.98 |  |  | (2, 34) = 0.62 |  |  | (2, 34) = 0.35 |  |
| Partial η^2^ | 0.01 |  |  | 0.01 |  |  | 0.06 |  |  | 0.04 |  |  | 0.02 |  |
| *P*-value | 0.82 |  |  | 0.93 |  |  | 0.39 |  |  | 0.54 |  |  | 0.71 |  |
| Post-hoc | - |  |  | - |  |  | - |  |  | - |  |  | - |  |
| **2-back** | ROI-1 | |  | ROI-2 | |  | ROI-3 | |  | ROI-4 | |  | ROI-5 | |
|  | Mean | SD |  | Mean | SD |  | Mean | SD |  | Mean | SD |  | Mean | SD |
| Control | 0.18 | 0.08 |  | 1.18 | 0.08 |  | 1.19 | 0.08 |  | 1.19 | 0.08 |  | 1.22 | 0.05 |
| Japanese | 1.17 | 0.05 |  | 1.17 | 0.05 |  | 1.18 | 5.00 |  | 1.19 | 0.06 |  | 1.21 | 0.04 |
| Tropical | 1.20 | 0.08 |  | 1.19 | 0.08 |  | 1.19 | 0.08 |  | 1.18 | 0.07 |  | 1.23 | 0.07 |
| *F*-value | (2, 34) = 0.98 |  |  | (2, 34) = 0.68 |  |  | (2, 34)= 0.09 |  |  | (2, 34) = 0.31 |  |  | (2, 34) = 1.19 |  |
| Partial η^2^ | 0.06 |  |  | 0.40 |  |  | 0.01 |  |  | 0.02 |  |  | 0.08 |  |
| *P*-value | 0.39 |  |  | 0.51 |  |  | 0.92 |  |  | 0.74 |  |  | 0.24 |  |
| Post-hoc | - |  |  | - |  |  | - |  |  | - |  |  | - |  |

EEG signals data from 32 sites were arranged into the five regions of interests (ROIs). ROI-1, left-frontal (FP1, F3, F7); ROI-2, right-frontal (FP2, F4, F8); ROI-3, left-posterior (P3, P7, O1); ROI-4, right-posterior (P4, P8, O2); ROI-5, midline (Fz, Cz, Pz).

Bold and italic - indicates statistically significant

Exposure, 5-min exposure**;** Stroop task, stroop color and word task; 1-back, 1-back task; 2-back, 2-back task; Control, control design; Japanese, Japanese design; Tropical, tropical design; SD, standard deviation
