# Supplementary material for: The effects of different designs of indoor biophilic greening on psychological and physiological responses and cognitive performance of office workers
Source: PLoS One. 2024 Jul 26;19(7):e0307934. doi: 10.1371/journal.pone.0307934 (PMC11280145; doi:10.1371/journal.pone.0307934)
Supplement: S5 Table — (DOCX) [file pone.0307934.s005.docx]

**S5 Table. Summary of the analysis of variance results on EEG alpha 2 powers in the 5-min exposure, Stroop, 1-back, and 2-back tasks.**

| **EEG alpha 2_Absolute power** | |  |  |  |  |  | |  | |  |  | |  | |  |  | |  | |  |  |
| --- | --- | --- | --- | --- | --- | --- | --- | --- | --- | --- | --- | --- | --- | --- | --- | --- | --- | --- | --- | --- | --- |
| **Exposure** | ROI-1 | |  | ROI-2 | | |  | | ROI-3 | | |  | | ROI-4 | | |  | | ROI-5 | | |
|  | Mean | SD |  | Mean | SD |  | | Mean | | SD |  | | Mean | | SD |  | | Mean | | SD |  |
| Control | 34.07 | 0.73 |  | 34.18 | 3.34 |  | | 37.14 | | 3.58 |  | | 38.51 | | 3.90 |  | | 36.34 | | 3.73 |  |
| Japanese | 32.85 | 0.75 |  | 32.95 | 3.65 |  | | 36.35 | | 4.19 |  | | 37.31 | | 4.16 |  | | 35.55 | | 3.55 |  |
| Tropical | 32.87 | 0.60 |  | 33.06 | 3.16 |  | | 36.12 | | 3.63 |  | | 37.10 | | 3.50 |  | | 35.37 | | 3.63 |  |
| *F*-value | (2, 34) = 4.08 |  |  | (2, 34) = 3.56 |  |  | | (2, 34) = 2.76 | |  |  | | (2, 34) = 3.41 | |  |  | | (2, 34) = 2.58 | |  |  |
| Partial η^2^ | 0.19 |  |  | 0.18 |  |  | | 0.14 | |  |  | | 0.17 | |  |  | | 0.13 | |  |  |
| *P*-value | ***0.03*** |  |  | ***0.04*** |  |  | | 0.08 | |  |  | | ***0.05*** | |  |  | | 0.09 | |  |  |
| Post-hoc | n.s |  |  | n.s |  |  | | - | |  |  | | ***Control > Tropical*** | | |  | | - | |  |  |
| **Stroop task** | ROI-1 | |  | ROI-2 | | |  | | ROI-3 | | |  | | ROI-4 | | |  | | ROI-5 | | |
|  | Mean | SD |  | Mean | SD |  | | Mean | | SD |  | | Mean | | SD |  | | Mean | | SD |  |
| Control | 33.46 | 4.80 |  | 33.19 | 4.06 |  | | 35.68 | | 5.13 |  | | 37.26 | | 5.80 |  | | 34.81 | | 5.00 |  |
| Japanese | 34.00 | 4.92 |  | 33.78 | 4.40 |  | | 36.19 | | 5.05 |  | | 37.13 | | 5.79 |  | | 35.22 | | 4.33 |  |
| Tropical | 34.44 | 3.95 |  | 34.87 | 4.14 |  | | 36.07 | | 4.97 |  | | 37.34 | | 4.96 |  | | 35.31 | | 4.86 |  |
| *F*-value | (2, 34) = 1.29 |  |  | (2, 34) = 4.38 |  |  | | (2, 34) = 0.39 | |  |  | | (2, 34) = 0.06 | |  |  | | (2, 34) = 0.40 | |  |  |
| Partial η^2^ | 0.07 |  |  | 0.21 |  |  | | 0.02 | |  |  | | 0.00 | |  |  | | 0.02 | |  |  |
| *P*-value | 0.29 |  |  | ***0.02*** |  |  | | 0.68 | |  |  | | 0.94 | |  |  | | 0.67 | |  |  |
| Post-hoc | - |  |  | ***Control < Tropical*** | |  | | - | |  |  | | - | |  |  | | - | |  |  |
| **1-back** | ROI-1 | |  | ROI-2 | | |  | | ROI-3 | | |  | | ROI-4 | | |  | | ROI-5 | | |
|  | Mean | SD |  | Mean | SD |  | | Mean | | SD |  | | Mean | | SD |  | | Mean | | SD |  |
| Control | 32.38 | 4.57 |  | 32.98 | 4.85 |  | | 33.37 | | 4.76 |  | | 34.57 | | 5.70 |  | | 32.75 | | 5.09 |  |
| Japanese | 32.68 | 4.54 |  | 33.48 | 4.55 |  | | 33.12 | | 5.13 |  | | 35.00 | | 5.03 |  | | 33.28 | | 4.46 |  |
| Tropical | 31.78 | 4.32 |  | 32.38 | 4.09 |  | | 33.55 | | 4.58 |  | | 33.30 | | 5.70 |  | | 32.30 | | 4.25 |  |
| *F*-value | (2, 34) = 0.67 |  |  | (2, 34) = 1.21 |  |  | | (2, 34) = 0.13 | |  |  | | (2, 34) = 1.79 | |  |  | | (2, 34) = 0.52 | |  |  |
| Partial η^2^ | 0.04 |  |  | 0.07 |  |  | | 0.01 | |  |  | | 0.10 | |  |  | | 0.03 | |  |  |
| *P*-value | 0.52 |  |  | 0.31 |  |  | | 0.88 | |  |  | | 0.18 | |  |  | | 0.60 | |  |  |
| Post-hoc | - |  |  | - |  |  | | - | |  |  | | - | |  |  | | - | |  |  |
| **2-back** | ROI-1 | |  | ROI-2 | | |  | | ROI-3 | | |  | | ROI-4 | | |  | | ROI-5 | | |
|  | Mean | SD |  | Mean | SD |  | | Mean | | SD |  | | Mean | | SD |  | | Mean | | SD |  |
| Control | 30.90 | 4.31 |  | 30.93 | 4.31 |  | | 31.79 | | 5.47 |  | | 32.55 | | 5.40 |  | | 31.36 | | 4.66 |  |
| Japanese | 30.67 | 4.47 |  | 30.77 | 4.06 |  | | 31.64 | | 5.73 |  | | 32.24 | | 5.57 |  | | 31.07 | | 4.76 |  |
| Tropical | 32.05 | 3.78 |  | 31.39 | 3.78 |  | | 32.56 | | 4.91 |  | | 34.14 | | 5.06 |  | | 32.28 | | 4.50 |  |
| *F*-value | (2, 34) = 02.51 |  |  | (2, 34) = 0.38 |  |  | | (2, 34) = 0.85 | |  |  | | (2, 34) = 2.19 | |  |  | | (2, 34) = 2.38 | |  |  |
| Partial η^2^ | 0.13 |  |  | 0.02 |  |  | | 0.05 | |  |  | | 0.11 | |  |  | | 0.12 | |  |  |
| *P*-value | 0.10 |  |  | 0.69 |  |  | | 0.44 | |  |  | | 0.13 | |  |  | | 0.11 | |  |  |
| Post-hoc | - |  |  | - |  |  | | - | |  |  | | - | |  |  | | - | |  |  |
|  |  |  |  |  |  |  | |  | |  |  | |  | |  |  | |  | |  |  |
| **EEG alpha 2_Relative power** | |  |  |  |  |  | |  | |  |  | |  | |  |  | |  | |  |  |
| **Exposure** | ROI-1 | |  | ROI-2 | | |  | | ROI-3 | | |  | | ROI-4 | | |  | | ROI-5 | | |
|  | Mean | SD |  | Mean | SD |  | | Mean | | SD |  | | Mean | | SD |  | | Mean | | SD |  |
| Control | 1.33 | 0.10 |  | 1.29 | 0.07 |  | | 1.29 | | 0.06 |  | | 1.32 | |  |  | | 1.30 | | 0.07 |  |
| Japanese | 1.30 | 0.11 |  | 1.27 | 0.10 |  | | 1.27 | | 0.77 |  | | 1.30 | |  |  | | 1.28 | | 0.08 |  |
| Tropical | 1.32 | 0.09 |  | 1.30 | 0.10 |  | | 1.28 | | 0.07 |  | | 1.30 | |  |  | | 1.29 | | 0.06 |  |
| *F*-value | (2, 34) = 1.79 |  |  | (2, 34) = 1.44 |  |  | | (2, 34) = 0.88 | |  |  | | (2, 34) = 2.22 | |  |  | | (2, 34) = 1.38 | |  |  |
| Partial η^2^ | 0.01 |  |  | 0.08 |  |  | | 0.05 | |  |  | | 0.12 | |  |  | | 0.08 | |  |  |
| *P*-value | 0.18 |  |  | 0.25 |  |  | | 0.43 | |  |  | | 0.13 | |  |  | | 0.27 | |  |  |
| Post-hoc | - |  |  | - |  |  | | - | |  |  | | - | |  |  | | - | |  |  |
| **Stroop task** | ROI-1 | |  | ROI-2 | | |  | | ROI-3 | | |  | | ROI-4 | | |  | | ROI-5 | | |
|  | Mean | SD |  | Mean | SD |  | | Mean | | SD |  | | Mean | | SD |  | | Mean | | SD |  |
| Control | 1.28 | 0.11 |  | 1.26 | 0.10 |  | | 1.27 | | 0.09 |  | | 1.30 | | 0.11 |  | | 1.29 | | 0.09 |  |
| Japanese | 1.26 | 0.11 |  | 1.27 | 0.11 |  | | 1.28 | | 0.10 |  | | 1.29 | | 0.10 |  | | 1.27 | | 0.09 |  |
| Tropical | 1.25 | 0.09 |  | 1.25 | 0.10 |  | | 1.26 | | 0.09 |  | | 1.27 | | 0.10 |  | | 1.27 | | 0.09 |  |
| *F*-value | (2, 34) = 0.85 |  |  | (2, 34) = 0.38 |  |  | | (2, 34) = 0.53 | |  |  | | (2, 34) = 1.00 | |  |  | | (2, 34) = 2.04 | |  |  |
| Partial η^2^ | 0.05 |  |  | 0.02 |  |  | | 0.03 | |  |  | | 0.06 | |  |  | | 0.11 | |  |  |
| *P*-value | 0.44 |  |  | 0.69 |  |  | | 0.60 | |  |  | | 0.38 | |  |  | | 0.15 | |  |  |
| Post-hoc | - |  |  | - |  |  | | - | |  |  | | - | |  |  | | - | |  |  |
| **1-back** | ROI-1 | |  | ROI-2 | | |  | | ROI-3 | | |  | | ROI-4 | | |  | | ROI-5 | | |
|  | Mean | SD |  | Mean | SD |  | | Mean | | SD |  | | Mean | | SD |  | | Mean | | SD |  |
| Control | 1.21 | 0.11 |  | 1.20 | 0.10 |  | | 1.21 | | 0.09 |  | | 1.24 | | 0.10 |  | | 1.22 | | 0.08 |  |
| Japanese | 1.19 | 0.06 |  | 1.19 | 0.06 |  | | 1.22 | | 0.08 |  | | 1.24 | | 0.09 |  | | 1.21 | | 0.07 |  |
| Tropical | 1.19 | 0.08 |  | 1.19 | 0.07 |  | | 1.20 | | 0.08 |  | | 1.22 | | 0.09 |  | | 1.21 | | 0.08 |  |
| *F*-value | (2, 34) = 0.62 |  |  | (2, 34) = 0.28 |  |  | | (2, 34) = 0.42 | |  |  | | (2, 34) = 1.06 | |  |  | | (2, 34) = 0.49 | |  |  |
| Partial η^2^ | 0.04 |  |  | 0.02 |  |  | | 0.03 | |  |  | | 0.06 | |  |  | | 0.03 | |  |  |
| *P*-value | 0.54 |  |  | 0.76 |  |  | | 0.66 | |  |  | | 0.36 | |  |  | | 0.62 | |  |  |
| Post-hoc | - |  |  | - |  |  | | - | |  |  | | - | |  |  | | - | |  |  |
| **2-back** | ROI-1 | |  | ROI-2 | | |  | | ROI-3 | | |  | | ROI-4 | | |  | | ROI-5 | | |
|  | Mean | SD |  | Mean | SD |  | | Mean | | SD |  | | Mean | | SD |  | | Mean | | SD |  |
| Control | 1.17 | 0.08 |  | 1.18 | 0.09 |  | | 1.21 | | 0.09 |  | | 1.21 | | 10.00 |  | | 1.21 | | 0.06 |  |
| Japanese | 1.17 | 0.08 |  | 1.18 | 0.08 |  | | 1.20 | | 0.08 |  | | 1.22 | | 0.10 |  | | 1.20 | | 0.07 |  |
| Tropical | 1.19 | 0.09 |  | 1.18 | 0.08 |  | | 1.20 | | 0.09 |  | | 1.21 | | 0.09 |  | | 1.21 | | 0.07 |  |
| *F*-value | (2, 34) = 0.83 |  |  | (2, 34) = 0.12 |  |  | | (2, 34) = 0.11 | |  |  | | (2, 34) = 0.08 | |  |  | | (2, 34) = 0.09 | |  |  |
| Partial η^2^ | 0.05 |  |  | 0.01 |  |  | | 0.01 | |  |  | | 0.01 | |  |  | | 0.02 | |  |  |
| *P*-value | 0.45 |  |  | 0.89 |  |  | | 0.90 | |  |  | | 0.92 | |  |  | | 0.75 | |  |  |
| Post-hoc | - |  |  | - |  |  | | - | |  |  | | - | |  |  | | - | |  |  |

EEG signals data from 32 sites were arranged into the five regions of interests (ROIs). ROI-1, left-frontal (FP1, F3, F7); ROI-2, right-frontal (FP2, F4, F8); ROI-3, left-posterior (P3, P7, O1); ROI-4, right-posterior (P4, P8, O2); ROI-5, midline (Fz, Cz, Pz).

Bold and italic - indicates statistically significant

Exposure, 5-min exposure**;** Stroop task, stroop color and word task; 1-back, 1-back task; 2-back, 2-back task; Control, control design; Japanese, Japanese design; Tropical, tropical design; SD, standard deviation; n.s., not significant
