# Supplementary material for: The effects of different designs of indoor biophilic greening on psychological and physiological responses and cognitive performance of office workers
Source: PLoS One. 2024 Jul 26;19(7):e0307934. doi: 10.1371/journal.pone.0307934 (PMC11280145; doi:10.1371/journal.pone.0307934)
Supplement: S6 Table — (DOCX) [file pone.0307934.s006.docx]

**S6 Table. Summary of the analysis of variance results on EEG beta 1 powers in the 5-min exposure, Stroop, 1-back, and 2-back tasks.**

| **EEG beta 1_Absolute power** | |  |  |  |  |  |  |  |  |  |  |  |  |  |
| --- | --- | --- | --- | --- | --- | --- | --- | --- | --- | --- | --- | --- | --- | --- |
| **Exposure** | ROI-1 | |  | ROI-2 | |  | ROI-3 | |  | ROI-4 | |  | ROI-5 | |
|  | Mean | SD |  | Mean | SD |  | Mean | SD |  | Mean | SD |  | Mean | SD |
| Control | 28.20 | 2.00 |  | 29.06 | 2.51 |  | 31.78 | 2.72 |  | 32.27 | 2.71 |  | 30.84 | 2.23 |
| Japanese | 27.94 | 1.97 |  | 28.49 | 2.36 |  | 31.50 | 2.82 |  | 31.80 | 2.94 |  | 30.70 | 2.16 |
| Tropical | 27.94 | 1.97 |  | 28.49 | 2.36 |  | 31.50 | 2.82 |  | 31.80 | 2.94 |  | 30.70 | 2.16 |
| *F*-value | (2, 34) = 0.77 |  |  | (2, 34) = 1.56 |  |  | (2, 34) = 0.44 |  |  | (2, 34) = 1.30 |  |  | (2, 34) = 0.22 |  |
| Partial η^2^ | 0,.04 |  |  | 0.08 |  |  | 0.03 |  |  | 0.07 |  |  | 0.00 |  |
| *P*-value | 0.47 |  |  | 0.22 |  |  | 0.65 |  |  | 0.29 |  |  | 0.80 |  |
| Post-hoc | - |  |  | - |  |  | - |  |  | - |  |  | - |  |
| **Stroop task** | ROI-1 | |  | ROI-2 | |  | ROI-3 | |  | ROI-4 | |  | ROI-5 | |
|  | Mean | SD |  | Mean | SD |  | Mean | SD |  | Mean | SD |  | Mean | SD |
| Control | 28.76 | 3.18 |  | 28.92 | 2.11 |  | 30.84 | 3.51 |  | 31.76 | 3.34 |  | 29.76 | 3.08 |
| Japanese | 29.40 | 3.73 |  | 29.31 | 3.05 |  | 31.19 | 3.17 |  | 31.58 | 3.57 |  | 30.30 | 2.72 |
| Tropical | 29.98 | 2.89 |  | 30.48 | 3.30 |  | 31.51 | 3.08 |  | 32.31 | 3.06 |  | 30.62 | 3.44 |
| *F*-value | (2, 34) = 1.81 |  |  | (2, 34) = 3.18 |  |  | (2, 34) = 0.90 |  |  | (2, 34) = 1.28 |  |  | (2, 34) = 1.17 |  |
| Partial η^2^ | 0.10 |  |  | 0.16 |  |  | 0.05 |  |  | 0.07 |  |  | 0.06 |  |
| *P*-value | 0.18 |  |  | 0.05 |  |  | 0.42 |  |  | 0.29 |  |  | 0.32 |  |
| Post-hoc | - |  |  | - |  |  | - |  |  | - |  |  | - |  |
| **1-back** | ROI-1 | |  | ROI-2 | |  | ROI-3 | |  | ROI-4 | |  | ROI-5 | |
|  | Mean | SD |  | Mean | SD |  | Mean | SD |  | Mean | SD |  | Mean | SD |
| Control | 29.03 | 3.26 |  | 29.63 | 3.49 |  | 29.99 | 3.58 |  | 30.55 | 3.60 |  | 29.37 | 3.78 |
| Japanese | 29.39 | 3.44 |  | 30.22 | 3.47 |  | 29.61 | 3.83 |  | 30.92 | 3.58 |  | 29.74 | 3.19 |
| Tropical | 28.67 | 2.76 |  | 29.23 | 2.89 |  | 30.26 | 3.06 |  | 29.62 | 3.61 |  | 29.32 | 2.88 |
| *F*-value | (2, 34)=0.57 |  |  | (2, 34)=1.29 |  |  | (2, 34)=0.43 |  |  | (2, 34)=2.12 |  |  | (2, 34)=0.40 |  |
| Partial η^2^ | 0.04 |  |  | 0.08 |  |  | 0.03 |  |  | 0.12 |  |  | 0.03 |  |
| *P*-value | 0.57 |  |  | 0.29 |  |  | 0.65 |  |  | 0.14 |  |  | 0.67 |  |
| Post-hoc | - |  |  | - |  |  | - |  |  | - |  |  | - |  |
| **2-back** | ROI-1 | |  | ROI-2 | |  | ROI-3 | |  | ROI-4 | |  | ROI-5 | |
|  | Mean | SD |  | Mean | SD |  | Mean | SD |  | Mean | SD |  | Mean | SD |
| Control | 28.31 | 3.42 |  | 28.38 | 3.32 |  | 29.20 | 4.71 |  | 29.47 | 3.94 |  | 28.32 | 3.62 |
| Japanese | 28.20 | 3.25 |  | 28.29 | 3.20 |  | 28.85 | 4.56 |  | 29.00 | 3.96 |  | 28.09 | 3.50 |
| Tropical | 29.08 | 2.52 |  | 28.95 | 2.80 |  | 29.77 | 3.55 |  | 30.81 | 3.60 |  | 29.04 | 3.37 |
| *F*-value | (2, 34) =1.35 |  |  | (2, 34) = 0.46 |  |  | (2, 34) = 0.80 |  |  | (2, 34) = 2.92 |  |  | (2, 34) = 2.26 |  |
| Partial η^2^ | 0.07 |  |  | 0.03 |  |  | 0.05 |  |  | 0.15 |  |  | 0.12 |  |
| *P*-value | 0.27 |  |  | 0.64 |  |  | 0.46 |  |  | 0.07 |  |  | 0.12 |  |
| Post-hoc | - |  |  | - |  |  | - |  |  | - |  |  | - |  |
|  |  |  |  |  |  |  |  |  |  |  |  |  |  |  |
| **EEG beta 1_Relative power** | |  |  |  |  |  |  |  |  |  |  |  |  |  |
| **Exposure** | ROI-1 | |  | ROI-2 | |  | ROI-3 | |  | ROI-4 | |  | ROI-5 | |
|  | Mean | SD |  | Mean | SD |  | Mean | SD |  | Mean | SD |  | Mean | SD |
| Control | 1.10 | 0.05 |  | 0.09 | 0.04 |  | 1.10 | 0.03 |  | 1.11 | 0.04 |  | 1.10 | 0.04 |
| Japanese | 1.10 | 0.04 |  | 1.10 | 0.04 |  | 1.10 | 0.03 |  | 1.11 | 0.03 |  | 1.10 | 0,03 |
| Tropical | 1.11 | 0.04 |  | 1.11 | 0.04 |  | 0.11 | 0.03 |  | 1.10 | 0.03 |  | 1.11 | 0,03 |
| *F*-value | (2, 34) = 0.93 |  |  | (2, 34) = 2.28 |  |  | (2, 34) = 0.54 |  |  | (2, 34) = 0.10 |  |  | (2, 34) = 0.66 |  |
| Partial η^2^ | 0.05 |  |  | 0.12 |  |  | 0.03 |  |  | 0.01 |  |  | 0.04 |  |
| *P*-value | 0.41 |  |  | 0.12 |  |  | 0.59 |  |  | 0.90 |  |  | 0.52 |  |
| Post-hoc | - |  |  | - |  |  | - |  |  | - |  |  | - |  |
| **Stroop task** | ROI-1 | |  | ROI-2 | |  | ROI-3 | |  | ROI-4 | |  | ROI-5 | |
|  | Mean | SD |  | Mean | SD |  | Mean | SD |  | Mean | SD |  | Mean | SD |
| Control | 1.10 | 0.04 |  | 1.10 | 0.05 |  | 1.10 | 0.04 |  | 1.11 | 0.05 |  | 1.11 | 0.05 |
| Japanese | 1.09 | 0.04 |  | 1.10 | 0.04 |  | 1.10 | 0.04 |  | 1.10 | 0.03 |  | 1.10 | 0.03 |
| Tropical | 1.09 | 0.04 |  | 1.09 | 0.04 |  | 1.10 | 0.05 |  | 1.10 | 0.04 |  | 1.10 | 0.04 |
| *F*-value | (2, 34) = 0.99 |  |  | (2, 34) = 1.02 |  |  | (2, 34) = 0.02 |  |  | (2, 34) = 0.36 |  |  | (2, 34) = 1.53 |  |
| Partial η^2^ | 0.06 |  |  | 0.06 |  |  | 0.00 |  |  | 2.00 |  |  | 0.08 |  |
| *P*-value | 0.38 |  |  | 0.37 |  |  | 0.98 |  |  | 0.70 |  |  | 0.23 |  |
| Post-hoc | - |  |  | - |  |  | - |  |  | - |  |  | - |  |
| **1-back** | ROI-1 | |  | ROI-2 | |  | ROI-3 | |  | ROI-4 | |  | ROI-5 | |
|  | Mean | SD |  | Mean | SD |  | Mean | SD |  | Mean | SD |  | Mean | SD |
| Control | 1.08 | 0.05 |  | 1.08 | 0.04 |  | 1.09 | 0.05 |  | 1.10 | 0.04 |  | 1.10 | 0.05 |
| Japanese | 1.08 | 0.03 |  | 1.08 | 0.03 |  | 1.09 | 0.03 |  | 1.10 | 0.04 |  | 1.09 | 0.03 |
| Tropical | 1.07 | 0.03 |  | 1.08 | 0.03 |  | 1.09 | 0.03 |  | 1.10 | 0.03 |  | 1.08 | 0.03 |
| *F*-value | (2, 34) = 0.43 |  |  | (2, 34) = 0.008 |  |  | (2, 34) = 0.26 |  |  | (2, 34) = 1.68 |  |  | (2, 34) = 1.76 |  |
| Partial η^2^ | 0.03 |  |  | 0.00 |  |  | 0.02 |  |  | 0.10 |  |  | 0.10 |  |
| *P*-value | 0.65 |  |  | 0.99 |  |  | 0.78 |  |  | 0.20 |  |  | 0.19 |  |
| Post-hoc | - |  |  | - |  |  | - |  |  | - |  |  | - |  |
| **2-back** | ROI-1 | |  | ROI-2 | |  | ROI-3 | |  | ROI-4 | |  | ROI-5 | |
|  | Mean | SD |  | Mean | SD |  | Mean | SD |  | Mean | SD |  | Mean | SD |
| Control | 1.07 | 0.04 |  | 1.08 | 0.03 |  | 1.11 | 0.04 |  | 1.10 | 0.04 |  | 1.10 | 0.03 |
| Japanese | 1.08 | 0.04 |  | 1.08 | 0.03 |  | 1.10 | 0.04 |  | 1.10 | 0.04 |  | 1.09 | 0.04 |
| Tropical | 1.08 | 0.05 |  | 1.08 | 0.04 |  | 1.10 | 0.04 |  | 1.10 | 0.05 |  | 1.09 | 0.04 |
| *F*-value | (2, 34) = 0.59 |  |  | (2, 34) = 0.01 |  |  | (2, 34) = 0.61 |  |  | (2, 34) = 0.04 |  |  | (2, 34) = 0.13 |  |
| Partial η^2^ | 0.03 |  |  | 0.00 |  |  | 0.04 |  |  | 0.00 |  |  | 0.01 |  |
| *P*-value | 0.56 |  |  | 0.94 |  |  | 0.55 |  |  | 0.97 |  |  | 0.88 |  |
| Post-hoc | - |  |  | - |  |  | - |  |  | - |  |  | - |  |

EEG signals data from 32 sites were arranged into the five regions of interests (ROIs). ROI-1, left-frontal (FP1, F3, F7); ROI-2, right-frontal (FP2, F4, F8); ROI-3, left-posterior (P3, P7, O1); ROI-4, right-posterior (P4, P8, O2); ROI-5, midline (Fz, Cz, Pz).

Exposure, 5-min exposure**;** Stroop task, stroop color and word task; 1-back, 1-back task; 2-back, 2-back task; Control, control design; Japanese, Japanese design; Tropical, tropical design; SD, standard deviation
