# Supplementary material for: The effects of different designs of indoor biophilic greening on psychological and physiological responses and cognitive performance of office workers
Source: PLoS One. 2024 Jul 26;19(7):e0307934. doi: 10.1371/journal.pone.0307934 (PMC11280145; doi:10.1371/journal.pone.0307934)
Supplement: S7 Table — (DOCX) [file pone.0307934.s007.docx]

**S7 Table. Summary of the analysis of variance results on EEG beta 2 powers in the 5-min exposure, Stroop, 1-back, and 2-back tasks.**

| **EEG beta 2_Absolute power** | |  |  |  |  |  |  |  |  |  |  |  |  |  |
| --- | --- | --- | --- | --- | --- | --- | --- | --- | --- | --- | --- | --- | --- | --- |
| **Exposure** | ROI-1 | |  | ROI-2 | |  | ROI-3 | |  | ROI-4 | |  | ROI-5 | |
|  | Mean | SD |  | Mean | SD |  | Mean | SD |  | Mean | SD |  | Mean | SD |
| Control | 26.70 | 2.17 |  | 27.83 | 2.30 |  | 30.28 | 2.50 |  | 30.32 | 2.39 |  | 29.13 | 1.99 |
| Japanese | 26.00 | 1.97 |  | 26.63 | 2.45 |  | 29.20 | 2.73 |  | 29.29 | 2.68 |  | 28.35 | 2.16 |
| Tropical | 25.52 | 2.00 |  | 26.36 | 2.61 |  | 28.76 | 1.88 |  | 29.01 | 2.18 |  | 28.07 | 2.50 |
| *F*-value | (2, 34) = 3.20 |  |  | (2, 34) = 4.96 |  |  | (2, 34) = 7.13 |  |  | (2, 34) = 5.40 |  |  | (2, 34) = 5.59 |  |
| Partial η^2^ | 0.16 |  |  | 0.23 |  |  | 0.30 |  |  | 0.24 |  |  | 0.25 |  |
| *P*-value | 0.05 |  |  | ***0.02*** |  |  | ***< 0.01*** |  |  | ***< 0.01*** |  |  | ***< 0.01*** |  |
| Post-hoc | - |  |  | ***Control > Japanese, Tropical*** | |  | ***Control > Tropical*** | |  | ***Control > Japanese, Tropical*** | |  | ***Control > Tropical*** | |
| **Stroop task** | ROI-1 | |  | ROI-2 | |  | ROI-3 | |  | ROI-4 | |  | ROI-5 | |
|  | Mean | SD |  | Mean | SD |  | Mean | SD |  | Mean | SD |  | Mean | SD |
| Control | 27.16 | 2.47 |  | 27.39 | 1.73 |  | 29.15 | 2.88 |  | 29.78 | 2.48 |  | 27.80 | 2.31 |
| Japanese | 27.71 | 3.63 |  | 27.48 | 2.91 |  | 28.81 | 2.91 |  | 29.10 | 3.20 |  | 27.98 | 2.31 |
| Tropical | 28.23 | 2.76 |  | 28.59 | 3.07 |  | 29.20 | 3.04 |  | 30.00 | 2.84 |  | 28.26 | 3.20 |
| *F*-value | (2, 34) = 0.99 |  |  | (2, 34) = 1.66 |  |  | (2, 34) = 0.40 |  |  | (2, 34) = 1.66 |  |  | (2, 34) = 00.47 |  |
| Partial η^2^ | 0.06 |  |  | 0.09 |  |  | 0.02 |  |  | 0.90 |  |  | 0.03 |  |
| *P*-value | 0.38 |  |  | 0.21 |  |  | 0.67 |  |  | 0.21 |  |  | 0.63 |  |
| Post-hoc | - |  |  | - |  |  | - |  |  | - |  |  | - |  |
| **1-back** | ROI-1 | |  | ROI-2 | |  | ROI-3 | |  | ROI-4 | |  | ROI-5 | |
|  | Mean | SD |  | Mean | SD |  | Mean | SD |  | Mean | SD |  | Mean | SD |
| Control | 29.03 | 3.26 |  | 29.63 | 3.49 |  | 29.99 | 3.58 |  | 30.55 | 3.60 |  | 29.37 | 3.78 |
| Japanese | 29.39 | 3.44 |  | 30.22 | 3.47 |  | 29.61 | 3.83 |  | 30.93 | 3.58 |  | 29.74 | 3.19 |
| Tropical | 28.67 | 2.76 |  | 29.23 | 2.89 |  | 30.26 | 3.06 |  | 29.62 | 3.60 |  | 29.32 | 2.88 |
| *F*-value | (2, 34) = 0.57 |  |  | (2, 34) = 1.29 |  |  | (2, 34) = 0.43 |  |  | (2, 34) = 2.12 |  |  | (2, 34) = 0.40 |  |
| Partial η^2^ | 0.04 |  |  | 0.08 |  |  | 0.03 |  |  | 0.12 |  |  | 0.03 |  |
| *P*-value | 0.57 |  |  | 0.29 |  |  | 0.65 |  |  | 0.14 |  |  | 0.67 |  |
| Post-hoc | - |  |  | - |  |  | - |  |  | - |  |  | - |  |
| **2-back** | ROI-1 | |  | ROI-2 | |  | ROI-3 | |  | ROI-4 | |  | ROI-5 | |
|  | Mean | SD |  | Mean | SD |  | Mean | SD |  | Mean | SD |  | Mean | SD |
| Control | 27.56 | 3.22 |  | 27.35 | 2.76 |  | 27.93 | 4.32 |  | 28.34 | 3.65 |  | 26.82 | 3.00 |
| Japanese | 27.08 | 2.89 |  | 27.14 | 3.16 |  | 27.17 | 3.85 |  | 27.12 | 3.04 |  | 26.38 | 2.89 |
| Tropical | 27.58 | 2.10 |  | 27.57 | 2.48 |  | 27.96 | 3.03 |  | 28.93 | 2.90 |  | 27.19 | 2.90 |
| *F*-value | (2, 34) = 0.46 |  |  | (2, 34) = 0.16 |  |  | (2, 34) = 0.78 |  |  | (2, 34) = 3.17 |  |  | (2, 34) = 2.63 |  |
| Partial η^2^ | 0.03 |  |  | 0.01 |  |  | 0.04 |  |  | 0.16 |  |  | 0.13 |  |
| *P*-value | 0.63 |  |  | 0.86 |  |  | 0.47 |  |  | 0.60 |  |  | 0.09 |  |
| Post-hoc | - |  |  | - |  |  | - |  |  | - |  |  | - |  |
|  |  |  |  |  |  |  |  |  |  |  |  |  |  |  |
| **EEG beta 2_Relative power** | |  |  |  |  |  |  |  |  |  |  |  |  |  |
| **Exposure** | ROI-1 | |  | ROI-2 | |  | ROI-3 | |  | ROI-4 | |  | ROI-5 | |
|  | Mean | SD |  | Mean | SD |  | Mean | SD |  | Mean | SD |  | Mean | SD |
| Control | 1.04 | 0.02 |  | 1.05 | 0.02 |  | 1.05 | 0.02 |  | 1.04 | 0.03 |  | 1.04 | 0.02 |
| Japanese | 1.02 | 0.02 |  | 1.03 | 0.02 |  | 1.02 | 0.02 |  | 1.02 | 0.02 |  | 1.02 | 0.02 |
| Tropical | 1.02 | 0.02 |  | 1.03 | 0.02 |  | 1.02 | 0.02 |  | 1.02 | 0.02 |  | 1.02 | 0.02 |
| *F*-value | (2, 34) = 7.19 |  |  | (2, 34) = 14.79 |  |  | (2, 34) = 31.45 |  |  | (2, 34) = 17.78 |  |  | (2, 34) = 28.35 |  |
| Partial η^2^ | 0.30 |  |  | 0.47 |  |  | 0.65 |  |  | 0.51 |  |  | 0.63 |  |
| *P*-value | ***< 0.01*** |  |  | ***< 0.01*** |  |  | ***< 0.01*** |  |  | ***< 0.01*** |  |  | ***< 0.01*** |  |
| Post-hoc | ***Control > Tropical*** | |  | ***Control > Japanese, Tropical*** | |  | ***Control > Japanese, Tropical*** | |  | ***Control > Japanese, Tropical*** | |  | ***Control > Japanese, Tropical*** | |
| **Stroop task** | ROI-1 | |  | ROI-2 | |  | ROI-3 | |  | ROI-4 | |  | ROI-5 | |
|  | Mean | SD |  | Mean | SD |  | Mean | SD |  | Mean | SD |  | Mean | SD |
| Control | 1.04 | 0.03 |  | 1.04 | 0.03 |  | 1.04 | 0.02 |  | 1.04 | 0.03 |  | 1.04 | 0.03 |
| Japanese | 1.02 | 0.03 |  | 1.03 | 0.03 |  | 1.02 | 0.03 |  | 1.02 | 0.02 |  | 1.01 | 0.03 |
| Tropical | 1.02 | 0.03 |  | 1.02 | 0.02 |  | 1.02 | 0.02 |  | 1.02 | 0.02 |  | 1.01 | 0.02 |
| *F*-value | (2, 34) = 4.40 |  |  | (2, 34) = 14.83 |  |  | (2, 34) = 20.95 |  |  | (2, 34) = 16.27 |  |  | (2, 34) = 18.27 |  |
| Partial η^2^ | 0.21 |  |  | 0.47 |  |  | 0.56 |  |  | 0.49 |  |  | 0.52 |  |
| *P*-value | ***0.02*** |  |  | ***< 0.01*** |  |  | ***< 0.01*** |  |  | ***< 0.01*** |  |  | ***< 0.01*** |  |
| Post-hoc | ***Control > Japanese, Tropical*** | |  | ***Control > Japanese, Tropical*** | |  | ***Control > Japanese, Tropical*** | |  | ***Control > Japanese, Tropical*** | |  | ***Control > Japanese, Tropical*** | |
| **1-back** | ROI-1 | |  | ROI-2 | |  | ROI-3 | |  | ROI-4 | |  | ROI-5 | |
|  | Mean | SD |  | Mean | SD |  | Mean | SD |  | Mean | SD |  | Mean | SD |
| Control | 1.04 | 0.03 |  | 1.04 | 0.23 |  | 1.04 | 0.03 |  | 1.04 | 0.04 |  | 1.03 | 0.03 |
| Japanese | 1.03 | 0.02 |  | 1.03 | 0.17 |  | 1.03 | 0.02 |  | 1.02 | 0.02 |  | 1.02 | 0.02 |
| Tropical | 1.02 | 0.02 |  | 1.03 | 0.02 |  | 1.03 | 0.01 |  | 1.02 | 0.02 |  | 1.02 | 0.02 |
| *F*-value | (2, 34) = 2.80 |  |  | (2, 34) = 1.16 |  |  | (2, 34) = 1.59 |  |  | (2, 34) = 3.34 |  |  | (2, 34) = 2.87 |  |
| Partial η^2^ | 0.15 |  |  | 0.07 |  |  | 0.09 |  |  | 0.17 |  |  | 0.15 |  |
| *P*-value | 0.08 |  |  | 0.33 |  |  | 0.22 |  |  | ***0.048*** |  |  | 0.07 |  |
| Post-hoc | - |  |  | - |  |  | - |  |  | *n.s.* |  |  | - |  |
| **2-back** | ROI-1 | |  | ROI-2 | |  | ROI-3 | |  | ROI-4 | |  | ROI-5 | |
|  | Mean | SD |  | Mean | SD |  | Mean | SD |  | Mean | SD |  | Mean | SD |
| Control | 1.04 | 0.04 |  | 1.05 | 0.30 |  | 1.06 | 0.03 |  | 1.06 |  |  | 1.04 | 0.03 |
| Japanese | 1.04 | 0.0.2 |  | 1.04 | 0.02 |  | 1.03 | 0.02 |  | 1.03 |  |  | 1.03 | 0.02 |
| Tropical | 1.03 | 0.02 |  | 1.03 | 0.02 |  | 1.03 | 0.01 |  | 1.03 |  |  | 1.02 | 0.02 |
| *F*-value | (2, 34) = 2.30 |  |  | (2, 34) = 2.57 |  |  | (2, 34) = 11.47 |  |  | (2, 34) = 7.48 |  |  | (2, 34) = 3.10 |  |
| Partial η^2^ | 0.12 |  |  | 0.13 |  |  | 0.40 |  |  | 0.31 |  |  | 0.15 |  |
| *P*-value | 0.12 |  |  | 0.09 |  |  | ***< 0.01*** |  |  | ***0.03*** |  |  | 0.06 |  |
| Post-hoc | - |  |  | - |  |  | ***Control > Japanese, Tropical*** | |  | ***Control > Japanese, Tropical*** | |  | - |  |

EEG signals data from 32 sites were arranged into the five regions of interests (ROIs). ROI-1, left-frontal (FP1, F3, F7); ROI-2, right-frontal (FP2, F4, F8); ROI-3, left-posterior (P3, P7, O1); ROI-4, right-posterior (P4, P8, O2); ROI-5, midline (Fz, Cz, Pz).

Bold and italic - indicates statistically significant

Exposure, 5-min exposure**;** Stroop task, stroop color and word task; 1-back, 1-back task; 2-back, 2-back task; Control, control design; Japanese, Japanese design; Tropical, tropical design; SD, standard deviation
